# Crystal structures of a bacterial dipeptidyl peptidase IV reveal a novel substrate recognition mechanism distinct from that of mammalian orthologues

**DOI:** 10.1038/s41598-018-21056-y

**Published:** 2018-02-09

**Authors:** Saori Roppongi, Yoshiyuki Suzuki, Chika Tateoka, Mayu Fujimoto, Saori Morisawa, Ippei Iizuka, Akihiro Nakamura, Nobuyuki Honma, Yosuke Shida, Wataru Ogasawara, Nobutada Tanaka, Yasumitsu Sakamoto, Takamasa Nonaka

**Affiliations:** 1 School of Pharmacy, Iwate Medical University, 2-1-1 Nishitokuta, Yahaba, Iwate 028-3694 Japan; 2 Department of Bioengineering, Nagaoka University of Technology, 1603-1 Kamitomioka, Nagaoka, Niigata 940-2188 Japan; 3 School of Pharmacy, Showa University, 1-5-8 Hatanodai, Shinagawa-ku, Tokyo 142-8555 Japan

## Abstract

Dipeptidyl peptidase IV (DPP IV, DPP4, or DAP IV) preferentially cleaves substrate peptides with Pro or Ala at the P1 position. The substrate recognition mechanism has been fully elucidated for mammalian DPP IV by crystal structure analyses but not for bacterial orthologues. Here, we report the crystal structures of a bacterial DPP IV (PmDAP IV) in its free form and in complexes with two kinds of dipeptides as well as with a non-peptidyl inhibitor at 1.90 to 2.47 Å resolution. Acyl-enzyme intermediates were observed for the dipeptide complexes of PmDAP IV, whereas tetrahedral intermediates were reported for the oligopeptide complexes of mammalian DPP IVs. This variation reflects the different structural environments of the active site Arg residues, which are involved in the recognition of a substrate carbonyl group, of mammalian and bacterial enzymes. A phylogenetic analysis revealed that PmDAP IV is a closer relative of dipeptidyl peptidases 8 and 9 (DPP8 and DPP9, DPP IV-family enzymes) than DPP IV. These results provide new insights into the substrate recognition mechanism of bacterial DAP IVs and may assist in the development of selective inhibitors for DAP IVs from pathogenic asaccharolytic bacteria, which utilise proteins or peptides as an energy source.

## Introduction

Peptidases catalyse the hydrolysis of peptide bonds. These enzymes are widely distributed in nature and are involved in a wide variety of biological functions^1^. Peptidases can be grouped according to the pattern of proteolysis as either endo- or exo-peptidases. Exopeptidases catalyse the removal of amino acids (or short peptides) from the end of a polypeptide chain, whereas endopeptidases cleave a peptide bond between nonterminal amino acids. According to International Union of Biochemistry and Molecular Biology (IUBMB) nomenclature, exopeptidases are divided into aminopeptidases, dipeptidases, dipeptidyl-peptidases, tripeptidyl-peptidases, carboxypeptidases and omega peptidases.

Dipeptidyl peptidase IV (DPP IV or DPP4, EC 3.4.14.5) is a homodimeric serine peptidase, each subunit consisting of approximately 700 amino acids, and is classified into the clan SC family S9 in the MEROPS data base^2^. This enzyme preferentially cleaves substrate peptides with Pro or Ala at the penultimate position of peptides [NH_2_-P2-P1(Pro/Ala)-//-P1′–P2′…]. DPP IV has been isolated from bacteria^3–11^, fungi^12,13^ and mammals^14–18^. In mammals, DPP IV is responsible for the degradation of incretins, such as GLP-1^19,20^, and plays a major role in glucose metabolism; thus, DPP IV is a well-known target of a new class of oral hypoglycaemics^21,22^. Mammalian DPP IV has also been identified as adenosine deaminase binding protein “ADA-bp”^23,24^ or T-cell activation antigen “CD26”. Several reviews of structural and functional studies of mammalian DPP IV and related enzymes have been published^25–27^.

In contrast, bacterial DPP IVs (in this paper, we designate bacterial DPP IV as DAP IV) play an important nutritional role in asaccharolytic bacteria, which utilise proteins or peptides as an energy source, cooperatively with other peptidases^28^. Because the DAP IV gene is found in many pathogenic bacteria, such as *Porphyromonas gingivalis* (which causes periodontitis)^29^ and *Stenotrophomonas maltophilia* (which causes opportunistic infections)^30^, structural information for a bacterial DAP IV would assist in the development of selective inhibitors for the DAP IVs of pathogenic bacteria.

Crystal structure analyses of mammalian DPP IVs have revealed that the enzyme is a homodimer (or a dimer of dimers for the pig enzyme), and each subunit comprises a C-terminal α/β hydrolase domain encompassing the enzymatic active site and an N-terminal, eight-bladed, β-propeller domain^31–34^. A conserved double-Glu motif^35^, Glu205-Glu206 (numbering for the human enzyme), forms salt bridges to the N-terminus (P2 residue) of a peptide substrate. The double-Glu motif is located on a short helix insertion in blade 4 of the β-propeller domain. The double-Glu motif also exists in a fibroblast activation protein α (FAP), a DPP IV-family enzyme^25^, which shares approximately 50% sequence identity to DPP IV and exhibits a DPP IV-like fold^36^. The side chain of Arg125, located in blade 2 of the β-propeller domain, is involved in the recognition of the carbonyl group of a peptide substrate^33,34^. The P1-binding pocket for smaller side chains (Pro or Ala) of the substrate is formed by the hydrophobic aromatic amino acids Tyr547, Tyr662 and Tyr666 from the α/β hydrolase domain, leaving no space for large substituents at that position^33,34^. Thus, the crystal structures of mammalian DPP IVs in complex with peptide substrates/products have been reported, but no crystal structure for bacterial DAP IV in complex with either inhibitor or reaction intermediates has yet been reported. The first bacterial DAP IV structure is that of ligand-free DAP IV from *S*. *maltophilia* (SmDAP IV) at 2.8 Å resolution^37^. Although the overall structure of SmDAP IV is similar to those of mammalian DPP IVs, insertions or deletions are found around the active site; for example, Arg125 (which is involved in the recognition of a substrate carbonyl group in mammalian DPP IV) is missing in SmDAP IV. In the absence of a crystal structure of a bacterial DAP IV complexed with an inhibitor (or reaction intermediates), a complete structural understanding of DAP IV-substrate interactions has thus far been impossible. This knowledge will be important to guide the structure-based design of novel selective inhibitors of pathogenic DAP IVs. A structural analysis of a bacterial DAP IV in complex with an inhibitor is clearly of broad interest. Recently, the crystal structure of DPP IV from *Porphyromonas gingivalis* (PgDPP IV) in a ligand-free form at 2.2 Å resolution was reported^38^. Unexpectedly, the overall structure and substrate-binding site of PgDPP IV are quite similar to those of human DPP IV rather than those of SmDAP IV. Arg125 in human DPP IV is conserved as Arg115 in PgDPP IV. This indicates that DPP IVs can be categorised into two groups: human DPP IV-type DPP IVs and SmDAP IV-type DAP IVs. PgDPP IV belongs to the human DPP IV group; therefore, we designate DPP IV from *P*. *gingivalis* as PgDPP IV rather than PgDAP IV.

Here, we report the crystal structures of DAP IV from *Pseudoxanthomonas mexicana* WO24 (PmDAP IV)^7,11^ in its free form and in complexes with two dipeptides (Lys-Pro and Ile-Pro) as well as with a non-peptidyl inhibitor at resolutions of 2.47, 1.90, 2.44 and 2.13 Å, respectively. The overall structure of PmDAP IV is clearly similar to that of SmDAP IV but differs from that of human DPP IV in several respects. The dipeptide complexes showed acyl-enzyme intermediates in the active site of PmDAP IV, whereas tetrahedral intermediates were reported for mammalian DPP IVs^34,39,40^. An active site Arg residue (Arg125 in human DPP IV) helps stabilise the tetrahedral intermediate in mammalian DPP IVs^34,39,40^ by interacting with the carbonyl group of the P1′ residue, whereas the structurally corresponding Arg residue in bacterial DAP IVs (Arg106 in PmDAP IV) does not play such a role. These observations provide new insights into the substrate recognition mechanism of bacterial DAP IVs.

## Results

### Overall structure of PmDAP IV

**Figure 1:**
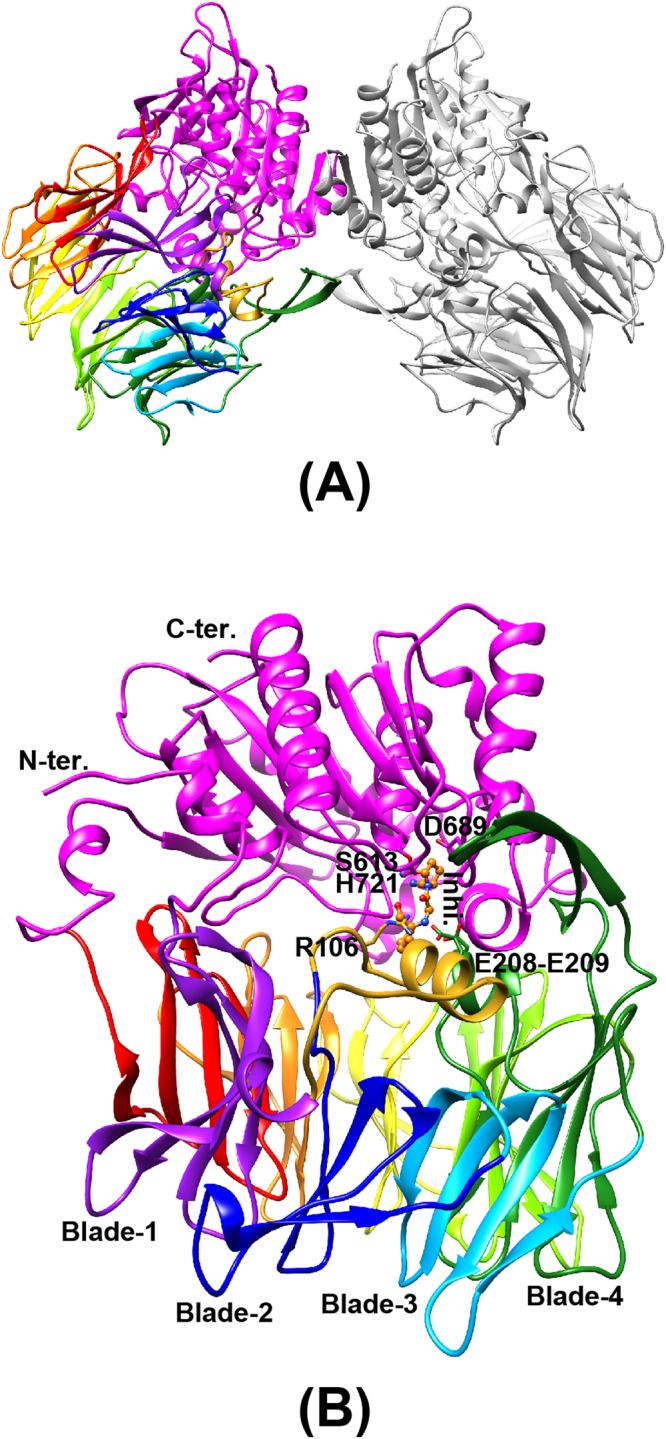
Three-dimensional structure of PmDAP IV. The catalytic domain is coloured in magenta. The β-propeller domain is coloured from purple (blade-1) to red (blade-8). A long insertion containing Arg106 located between blade-1 and blade-2, which was disordered in the ligand-free form, is shown in gold. (**A**) Dimeric structure of PmDAP IV. (**B**) PmDAP IV subunit. The catalytic triad “Ser613, Asp689, and His721”, the double-Glu motif “Glu208-Glu209”, Arg106 located between blade-1 and blade-2, a bound inhibitor molecule, and blades 1 to 4 are labelled.

The PmDAP IV enzyme forms a homodimer, with each subunit consisting of 724 residues (Ala22-Pro745) and a molecular weight of approximately 160 kDa (Fig. 1A). The N-terminal region of PmDAP IV has a typical signal sequence of gram-negative bacteria and hence PmDAP IV exists as a soluble form in the periplasm^11^, while the mammalian DPP IV and FAP are type II transmembrane proteins^25^. The crystal structures of PmDAP IV in the ligand-free form and in complexes with dipeptides (Lys-Pro or Ile-Pro) and a non-peptidyl inhibitor (Inhibitor-1c) were determined at resolutions of 2.47, 1.90, 2.44 and 2.13 Å, respectively (Tables 1 and 2). Representative electron density maps of the bound peptides and inhibitor are shown in Figure S1. The electron densities of the P2 side chains (Lys and Ile) are poorer than the P1-Pro residue covalently attached to the Oγ atom of Ser613 (Figures S1A and S1C). These are consistent with the results of refinement that the temperature factors of the bound ligand atoms are higher for some flexible groups away from the acyl-intermediate bond. This is also true for the inhibitor complex (Figure S1D). In the present crystal structure analysis of the ligand-free PmDAP IV, the asymmetric unit comprises two dimers of PmDAP IV. Dimerisation is also observed in the crystal structure of mammalian DPP IVs^31–34^. The protruding anti-parallel β-sheet dimerisation interface reported for mammalian DPP IVs is also observed for PmDAP IV. This type of inter-subunit interaction has been found among several crystal forms of PmDAP IV (Table 1), indicating that such dimer formation is the natural state of PmDAP IV. The protomer of PmDAP IV consists of two domains separated by a deep cleft (Fig. 1B). One domain, containing the α/β hydrolase fold harbouring the Asp-His-Ser catalytic triad, is responsible for catalysis; the second, β-propeller domain is the regulatory domain that is necessary for exopeptidase activity. A topology diagram of the PmDAP IV protomer is shown in Figure S2.

**Table 1:** Data collection statistics for PmDAP IV.

Data set	Free	Inhibitor-1c	Ile-Pro	Lys-Pro
Facility Beamline	Photon Factory BL17A	Photon Factory BL1A	Photon Factory BL17A	Photon Factory BL17A
Wavelength (Å)	0.9800	1.1000	0.9788	0.9800
Detector	ADSC Q315	PILATUS-2M	ADSC Q270	ADSC Q270
Crystal-to-detector distance (mm)	395.3	242.2	368.3	250.1
Rotation angle per image (°)	0.5	0.3	0.15	0.15
Total rotation range (°)	190.0	160.2	190.05	200.10
Exposure time per image (sec)	5.00	2.50	0.75	1.00
Space group	*P*2_1_2_1_2	*P*2_1_2_1_2_1_	*P*2_1_2_1_2_1_	*P*1
Cell dimensions				
*a* (Å) *b* (Å) *c* (Å)	149.73 326.19 71.00	59.88 120.22 231.89	119.88 120.12 262.53	88.66 104.49 112.84
α (°) β (°) γ (°)	90 90 90	90 90 90	90 90 90	67.42 68.83 65.46
Number of molecules per ASU	4	2	4	4
Mosaicity (°)	0.175	0.124	0.174	0.111
Resolution (Å) (outer shell)	2.47 (2.51–2.47)	2.13 (2.17–2.13)	2.44 (2.48–2.44)	1.90 (2.00–1.90)
No. of observed reflections	939,942 (30,274)	540,380 (22,266)	931,829 (19,145)	540,678 (59,278)
No. of unique reflections	125,591 (6,043)	93,976 (4,211)	137,770 (5,748)	245,086 (27,199)
Completeness (%)	99.9 (97.5)	99.3 (90.8)	97.5 (82.1)	94.2 (71.5)
Redundancy	7.5 (5.0)	5.8 (5.3)	6.8 (3.3)	2.2 (2.2)
*I* / σ_(*I*)_	14 (2.0)	9.0 (2.0)	14.4 (2.1)	6.5 (2.0)
CC_half_	0.998 (0.643)	0.994 (0.610)	0.998 (0.880)	0.989 (0.781)
*R*_merge_ (*I*)	0.125 (0.834)	0.140 (0.876)	0.087 (0.413)	0.082 (0.316)
*R*_meas_ (*I*)	0.135 (0.932)	0.154 (0.972)	0.093 (0.490)	0.110 (0.425)
*B*-factor (Å^2^)	31.8	29.1	39.8	19.2

**Table 2:** Refinement statistics for PmDAP IV.

Data set	Free	Inhibitor-1c	Ile-Pro	Lys-Pro
PDB ID	5YP1	5YP2	5YP3	5YP4
Resolution range (Å)	40.00–2.47	40.00–2.13	40.00–2.44	40.00–1.90
Completeness (%)	99.78	99.23	97.21	94.17
No. of reflections				
working set test set	119,191 6,289	89,236 4,652	130,819 6,835	232,846 12,222
*R*-factor	0.218	0.178	0.238	0.164
Free *R*-factor	0.284	0.226	0.286	0.213
No. of protein atoms (avg. *B*-factors (Å^2^))	22,059 (45.4)	11,320 (32.5)	22,640 (44.4)	22,270 (22.2)
No. of ligand atoms (avg. *B*-factors (Å^2^))	—	40 (2 × 20) (24.5)	60 (4 × 15) (25.7)	65 (3 × 16 + 1 × 17) (20.7)
No. of glycerol atoms (avg. *B*-factors (Å^2^))	6 (1 × 6) (48.0)	18 (3 × 6) (46.0)	6 (1 × 6) (52.3)	126 (21 × 6) (39.2)
No. of water molecules (avg. *B*-factors (Å^2^))	598 (33.6)	700 (33.5)	452 (31.3)	2,877 (31.3)
Ramachandran plot statistics				
favored (%) allowed (%) outlier (%)	2,617 (93.2) 164 (5.8) 27 (1.0)	1,392 (96.4) 52 (3.6) 0 (0.0)	2,710 (93.8) 172 (6.0) 6 (0.2)	2,747 (97.1) 82 (2.9) 0 (0.0)
RMSD				
bonds (Å) angles (°)	0.01 1.70	0.02 1.84	0.01 1.77	0.02 1.90

### Catalytic domain

**Figure 2:**
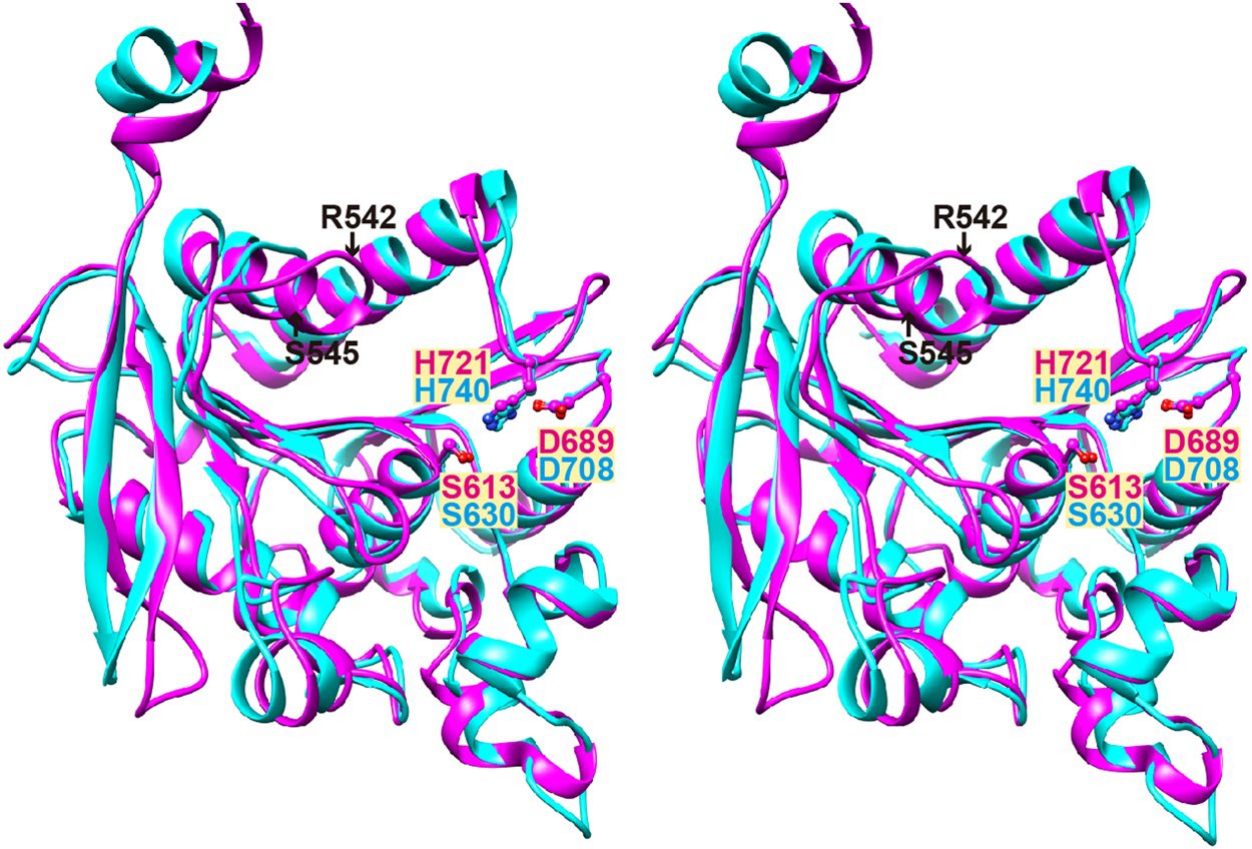
Wall-eyed stereo view showing a superposition of the catalytic domains of PmDAP IV (magenta) and human DPP IV (cyan). The catalytic triads of these enzymes are shown in ball-and-stick format. Each enzyme was superimposed with respect to the structurally equivalent 212 Cα atom pairs of the respective catalytic domains.

The catalytic domain includes residues 22–33 and 474–745, and forms an α/β hydrolase fold comprising a central eight-stranded β-sheet sandwiched by thirteen α-helices. The α/β hydrolase fold is characteristic of clan SC peptidases^2^, such as prolyl oligopeptidase (S9 family), carboxypeptidase Y (S10 family), Xaa-Pro dipeptidyl peptidase (S15 family), Pro-Xaa carboxypeptidase (S28 family), and prolyl aminopeptidase (S33 family). The catalytic domain of human DPP IV can be superimposed onto that of PmDAP IV (Fig. 2). The serine peptidase catalytic triads^41^, His721, Asp689 and Ser613 in PmDAP IV and His740, Asp708 and Ser630 in human DPP IV, are almost completely superimposable, with a root mean square (rms) deviation between the two structures of 0.95 Å for 212 structurally equivalent Cα atoms, which had 31% sequence identity for that region. Similarly, the rms deviation between the catalytic domains of PmDAP IV and SmDAP IV is 0.60 Å for 264 structurally equivalent Cα atoms, which had 77% sequence identity for that region.

### The β–propeller domain

**Figure 3:**
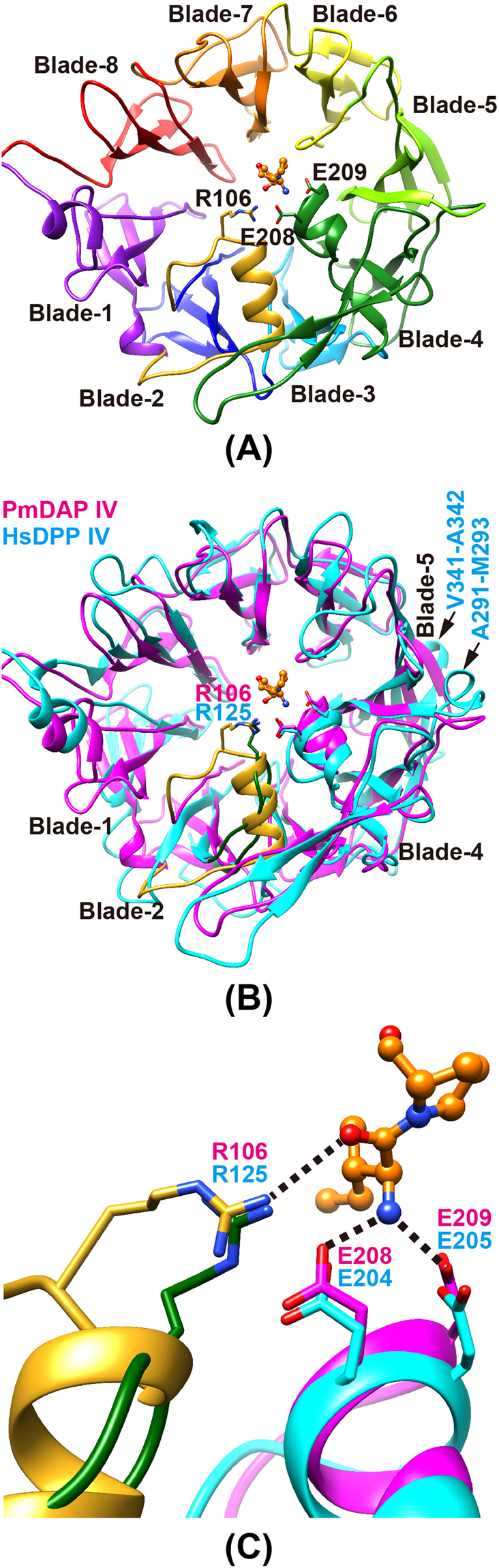
The β-propeller domain of PmDAP IV. (**A**) The β-propeller domain of PmDAP IV. A long insertion containing Arg106 located between blade-1 and blade-2, which was disordered in the ligand-free form, is shown in gold. A bound dipeptide, Ile-Pro, is shown in orange. (**B**) A superposition of the β-propeller domain of PmDAP IV (magenta) and that of human DPP IV (cyan). A hairpin loop containing Arg125 located between strands 2 and 3 of blade-2 in human DPP IV is shown in green. Exposed loops involved in interaction with other proteins in human DPP IV (Ala291-Met293 and Val341-Ala342) are labelled. (**C**) A detailed view of the conformations of Arg residues that are involved in the recognition of the P2-C = O group, of PmDAP IV and of human DPP IV.

The β–propeller domain forms a funnel-shaped structure comprising eight blades, each of which comprises four anti-parallel β–strands, except for blade 4 (Figs 3 and S2), which contains two additional components. One is a short helix between the first and second strands of blade 4. This helix contains a double-Glu motif, Glu208-Glu209, which forms salt bridges to the N-terminus of a peptide substrate (Fig. 3A). The other additional component in blade 4 is a protruding anti-parallel β-sheet between the second and third strands of blade 4 (Figure S2). The anti-parallel β-sheet acts as a dimerisation interface (Fig. 1A). A hairpin loop between the second and third strands of blade 2 containing Arg125 in human DPP IV, which can interact with the carbonyl group of a substrate peptide^33,34^, is replaced by a short turn in PmDAP IV (Fig. 3B). Interestingly, however, the side chain of Arg106 (located between blades 1 and 2 in PmDAP IV), which was disordered in the ligand-free form but was well defined in the peptide/inhibitor complexes (described later), appears to play a role equivalent to that of Arg125 in human DPP IV (Fig. 3C). Inter-domain contacts between the catalytic and β–propeller domains are observed for blades 4 to 8 of the β–propeller domain and the catalytic domain. Blades 1 to 3 of the β–propeller domain have less contact with the catalytic domain (Fig. 1B) and appear to be more flexible than blades 4 to 8. The rms deviation between the β–propeller domains of PmDAP IV and human DPP IV is 1.19 Å for 151 structurally equivalent Cα atoms, which had 19% sequence identity for that region, and the rms deviation between the β–propeller domains of PmDAP IV and SmDAP IV is 0.82 Å for 333 structurally equivalent Cα atoms, which had 73% sequence identity for that region. Thus, the structures of the β–propeller domains of PmDAP IV and SmDAP IV are somewhat different from that of the human enzyme.

### Conformational difference between the ligand-free and dipeptide/inhibitor-bound forms

**Figure 4:**
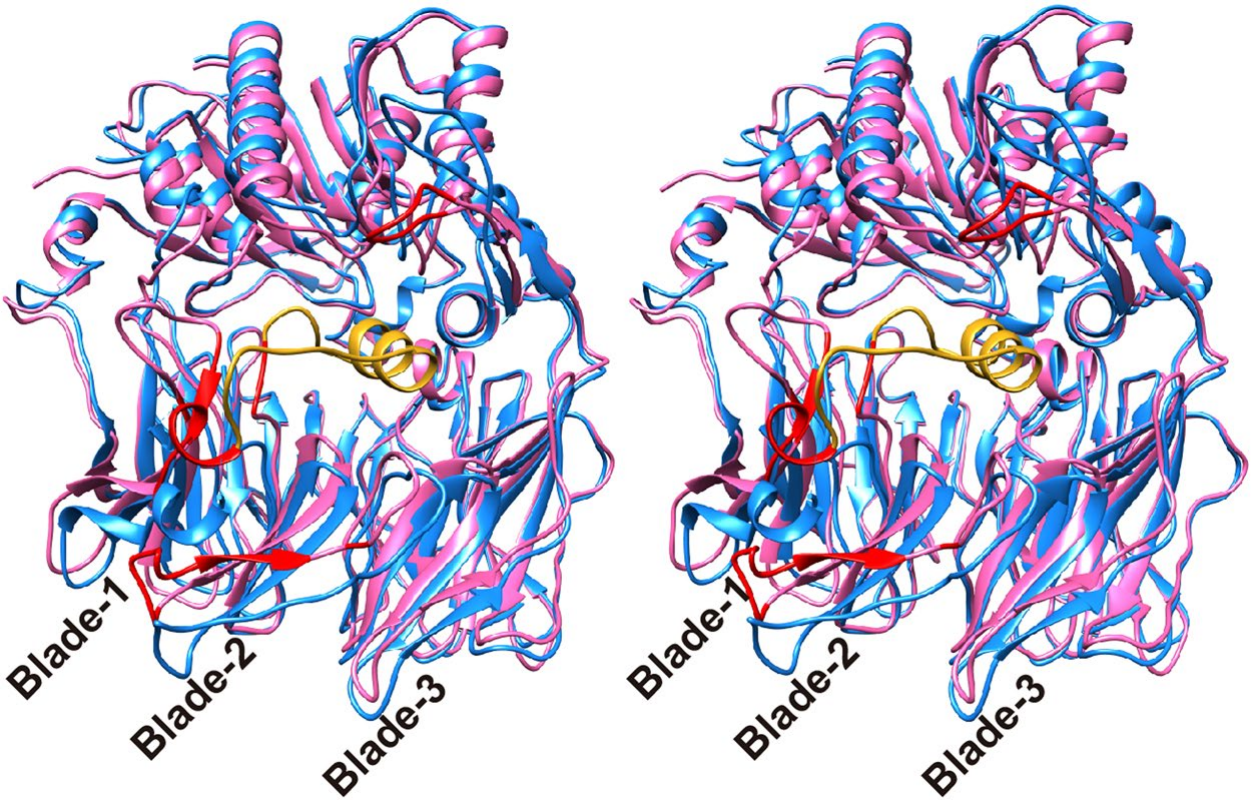
Wall-eyed stereo view showing a conformational difference of PmDAP IV. Ligand-free (cyan) and dipeptide-bound (magenta) forms of PmDAP IV are shown. Least-square fitting was performed with respect to all Cα atoms of each molecule. Cα-Cα deviations larger than 5 Å are shown in red for the dipeptide-bound form. A long insertion containing Arg106 located between blade-1 and blade-2, which was disordered in the ligand-free form, is shown in gold.

A comparison of the crystal structure of the ligand-free PmDAP IV with those of dipeptide/inhibitor-bound forms of PmDAP IV showed that the large cleft between the catalytic and β–propeller domains was relatively closed for the peptide/inhibitor-bound forms (Fig. 4). Larger conformational differences were observed mainly for blades 1 and 2 and the β-hairpin region of a protruding anti-parallel β-sheet between the second and third strands of blade 4. The closed conformation was observed for all crystallographically independent subunits of the dipeptide/inhibitor-bound complex crystals, in which the asymmetric units of the dipeptide and inhibitor complex crystals contain two and one dimer(s) of PmDAP IV, respectively (Table 1). Furthermore, the disordered loop region in the ligand-free form (residues 90–109 containing Arg106; Fig. 4) was well defined and formed a short helix in the dipeptide/inhibitor-bound complexes (Figure S3). Interestingly, one of the four subunits in the asymmetric unit of the ligand-free form exhibited a relatively closed form, similar to those observed for the dipeptide/inhibitor-bound forms, and the disordered loop region (residues 90–109) was well defined.

### Acyl-enzyme intermediate

A dipeptide Lys-Pro complex was obtained by co-crystallisation of PmDAP IV with the tripeptide Lys-Pro-Tyr. Clear continuous electron density was observed for the first two residues of the tripeptide (Figure S1A), and no clear electron density was observed for the last residue, Tyr. Because the PmDAP IV enzyme reaction occurs in the solution used for crystallisation, the Lys-Pro-Tyr tripeptide acts as the substrate, and the reaction products are the N-terminal Lys-Pro (P2-P1) and the C-terminal Tyr (P1′). While the N-terminal product Lys-Pro remains at the active site, the C-terminal product Tyr dissociates from the active site. Interestingly, we observed that the tripeptide was cleaved but remained trapped as an acyl-enzyme intermediate. The Oγ atom of Ser613 was found in close contact (1.4 Å) with the carbonyl carbon of the scissile bond. To further explore our observation that the N-terminal dipeptide product was trapped as an acyl-enzyme intermediate, the dipeptide and the Oγ atom of Ser613 were omitted from the model, and the structure was refined. The resultant omit electron density maps showed continuous electron density between the Oγ atom of Ser613 and the carbonyl carbon of the scissile bond (Figure S1A). The asymmetric unit was composed of four independent PmDAP IV subunits; in three of the four subunits, the dipeptides were trapped as the acyl-enzyme intermediate, and in the remaining subunit, the hydrolysed dipeptide product (NH_2_-Lys-Pro-COOH) was observed (Figure S1B). Similarly, co-crystallisation of PmDAP IV with the tripeptide Ile-Pro-Ile, a well-known peptidyl DPP IV inhibitor, diprotin A^42,43^, resulted in the dipeptide Ile-Pro complex being trapped as an acyl-enzyme intermediate (Figure S1C). In the Ile-Pro complex, acyl-enzyme intermediates were observed for all four subunits in the asymmetric unit.

### Dipeptide complex

**Figure 5:**
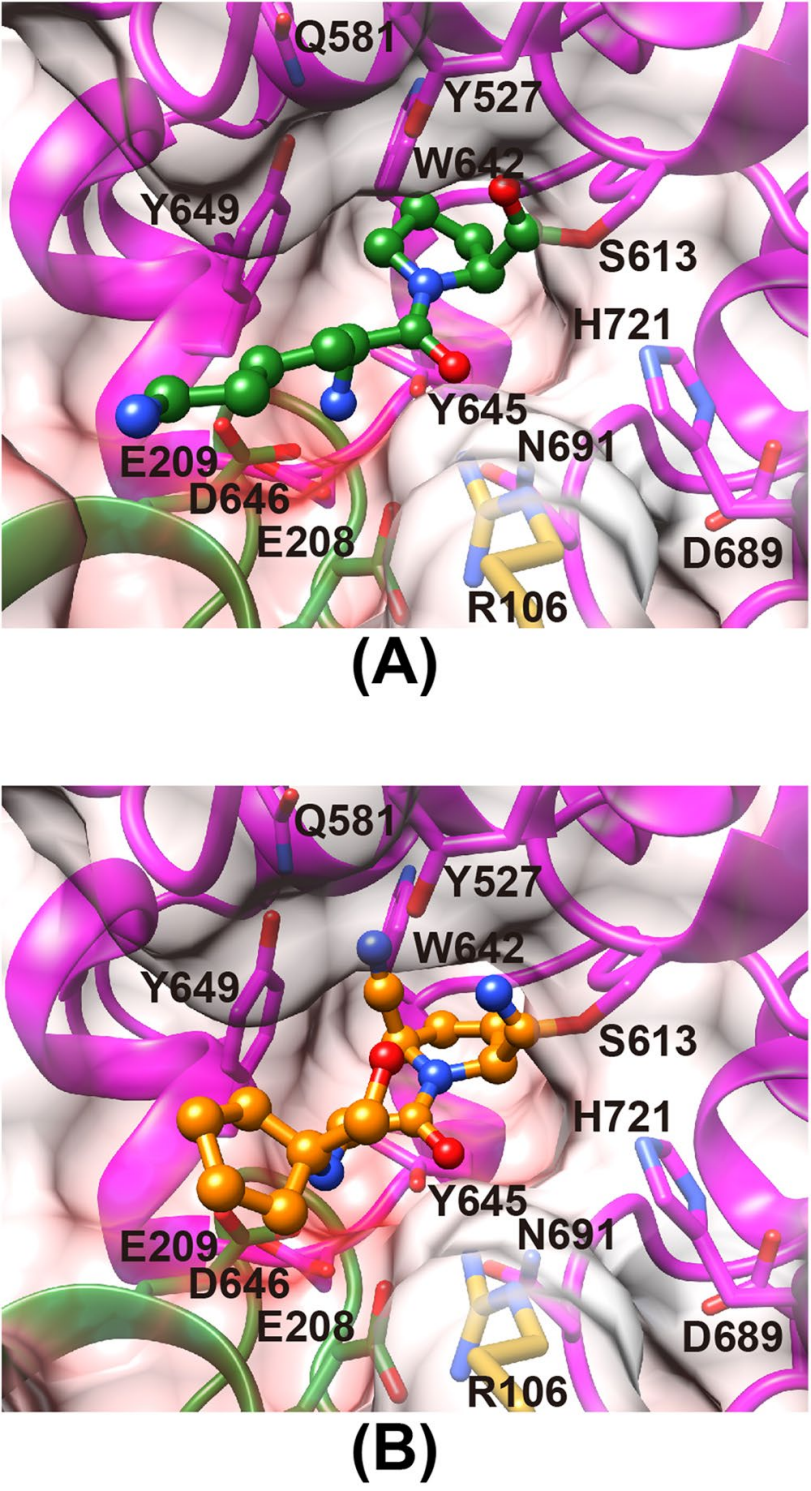
The mode of peptide/inhibitor binding in PmDAP IV. Residues belonging to the catalytic domain and blade 4 of the β-propeller domain are coloured magenta and green, respectively. The side chain of Arg106 is shown in gold. The surfaces are coloured in blue and red by a coulombic surface colouring to indicate positive and negative charges, respectively, from 30 kT to -30 kT. (**A**) Lys-Pro (green) complex. (**B**) Inhibitor-1c (orange) complex.

The dipeptide (Lys-Pro or Ile-Pro) complex structures clearly show the molecular basis for the peptide recognition mechanism of PmDAP IV. For simplicity, the following description refers primarily to subunit A of the 1.90-Å-resolution structure of the Lys-Pro complex of PmDAP IV. The bound dipeptide was found in the inter-domain cleft (Fig. 5A). PmDAP IV hydrolyses peptides from the N-terminus of oligopeptides, cleaving dipeptide units (NH_2_-P2-P1-COOH) when the second P1 residue is Pro or Ala. To act as dipeptidyl aminopeptidase, PmDAP IV must fix the N-terminus of the substrate peptide in position. This task is performed primarily by the side chains of a double-Glu motif (Glu208-Glu209), which forms part of a short helix between the first and second strands of blade 4 of the β–propeller domain. The double-Glu motif forms salt bridges to the N-terminus of the bound dipeptide. In addition, the side chain of Arg106, which is located between blades 1 and 2 of the β-propeller domain, forms a hydrogen bond with the carbonyl group of the P2 residue and also forms a salt bridge with the side chain of Glu208. The side chain of Asn691 also forms a hydrogen bond with the carbonyl group of the P2 residue. In the ligand-free form of PmDAP IV, residues 90–109, which are located between blades 1 and 2 of the β-propeller domain, were disordered. The corresponding region in SmDAP IV is also disordered^37^. In the dipeptide complex of PmDAP IV, the electron density of residues 90–109 was well defined and was interpreted as a short helix (Figure S3). The P1 Pro residue is accommodated in the S1 pocket, which comprises the side chains of Asn614, Val639, Trp642, Tyr645, Tyr649 and Val692. The side chain of the P2 Lys residue has less interaction with PmDAP IV and is surrounded by water molecules in the active site cleft. The carbonyl oxygen of the P1 residue is accommodated in the oxyanion hole and stabilised by hydrogen bonds with the main-chain imino group of Asn614 and the side-chain OH group of Tyr527. Similarly, an important role of the oxyanion hole (comprising the main-chain NH of Tyr631 and the side-chain OH of Tyr547) in the catalytic mechanism of human DPP IV has been reported^44^.

### Inhibitor complex

In addition to the dipeptide complex described above, a non-peptidyl inhibitor complex also shows the structural feature of the active site of PmDAP IV (Fig. 5B). The inhibitor used in this study is “Inhibitor-1c”, 1-({[1-(hydroxymethyl)cyclopentyl]amino}-acetyl)pyrrolidine-2,5-*cis*-dicarbonitrile, which is known to be an achiral inhibitor of mammalian DPP IV^45^ and is commercially available as a hydrochloride salt (Figure S4). The unbiased omit electron density map (Figure S1D) suggested that one nitrile group of the inhibitor is attacked by Ser613 to form a covalent bond. A similar covalent bond is also reported for the crystal structure analysis of human DPP IV in a complex with Inhibitor-1c, and imidate formation is confirmed by ^13^C-NMR^45^. The triple bond moiety of the other nitrile group stacks with the side chain of Tyr649. The pyrrolidine ring is accommodated in the S1 pocket (Asn614, Val639, Trp642, Tyr645, Tyr649 and Val692). The inhibitor moiety corresponding to a peptide P2 backbone is recognised by interactions from the side chains of Glu208 and Glu209 to the secondary amine group and from the side chains of Arg106 and Asn691 to the carbonyl group. The cyclopentane ring of the inhibitor has less contact with the active site, and this finding is consistent with the poorer electron density observed for the cyclopentane moiety compared with the pyrrolidine ring moiety (Figure S1D).

### Site-directed mutagenesis studies of PmDAP IV

To test the roles of Arg106 in substrate binding by PmDAP IV, we replaced the arginine residue with alanine or lysine and tested the enzymatic activity of the mutant proteins on a synthetic substrate, Gly-Pro-MCA (Table 3). The mutation of Arg106 to Ala resulted in a significant loss of activity (approximately 14% of the wild-type enzyme activity). This result indicates that Arg106 in PmDAP IV plays an important role in the recognition of the substrate peptide. Interestingly, the mutation of Arg106 to Lys also resulted in moderately decreased activities against the synthetic substrate (approximately 37% of the wild-type enzyme activity). These results indicate that both the positive charge and the strict binding mode provided by the side chain at position 106 are essential for the interaction with the carbonyl group of the substrate peptide and with the side chain of Glu208, as observed in the wild-type enzyme (Figs 3C and 5A).

**Table 3:** Kinetic parameters of PmDAP IVs and human DPP IV for the synthetic substrate Gly-Pro-MCA.

Enzyme	Specific activity (U mg^−1^)	Relative activity (%)	*k*_cat_ (sec^−1^)	*K*_m_ (µM)	*k*_cat_/*K*_m_ (sec^−1^ µM^−1^)
PmDAP IV wild-type	14.4 ± 0.3^#^	100	19.2 ± 0.4	19.4 ± 0.8	0.989 ± 0.035
PmDAP IV R106A	2.04 ± 0.05	14.2	2.72 ± 0.07	26.8 ± 0.9	0.102 ± 0.001
PmDAP IV R106K	5.36 ± 0.09	37.2	7.14 ± 0.12	34.5 ± 1.0	0.207 ± 0.003
Human DPP IV	4.13 ± 0.12	—	35.3 ± 1.0	37.1 ± 1.6	0.952 ± 0.013

^#^Standard deviation obtained from three independent experiments.

### Inhibitor sensitivities of PmDAP IV

To examine whether there is a significant difference in substrate recognition mechanism between PmDAP IV and mammalian DPP IV, the inhibitory activities of DPP IV inhibitors, also known as gliptins (Figure S5), against PmDAP IV and human DPP IV were evaluated (Table 4). Gliptins have been categorised into three classes according to their binding subsites^46^. Class 1 gliptins bind only to S1 and S2 subsites; class 2 bind only to S2, S1, S1′ and S2′ subsites; and class 3 bind only to S2, S1 and S2-extensive subsites. Our results showed that class 2 gliptins (linagliptin and trelagliptin) have significantly weaker inhibitory activities against PmDAP IV than do class 1 and 3 gliptins (Table 4). In contrast, class 2 gliptins have inhibitory activities against human DPP IV that are comparable to or stronger than class 1 and 3 gliptins.

**Table 4:** *K*_i_ and IC_50_ values of PmDAP IV and human DPP IV for several gliptins.

Classification	Compound	PmDAP IV		Human DPP IV	
		*K*_i_ (µM)	IC_50_ (µM)	*K*_i_ (nM)	IC_50_ (nM)
Class1	Vildagliptin	3.07 ± 0.39^#^	18.8 ± 2.4	2.18 ± 0.25	8.04 ± 0.91
	Saxagliptin	1.85 ± 0.13	11.1 ± 0.8	0.907 ± 0.013	3.35 ± 0.05
Class2	Linagliptin	30.0 ± 1.16	184 ± 7	0.00981 ± 0.00037	0.0362 ± 0.0014
	Trelagliptin	ND*	ND*	0.340 ± 0.006	1.26 ± 0.02
Class3	Sitagliptin	4.00 ± 0.59	24.6 ± 3.6	3.31 ± 0.22	12.2 ± 0.8
	Teneligliptin	2.34 ± 0.09	14. 3 ± 0.5	0.461 ± 0.004	1.70 ± 0.02
	Anagliptin	3.99 ± 0.07	24.5 ± 0.4	10.5 ± 0.6	38.6 ± 2.2
	Omarigliptin	23.8 ± 3.8	146 ± 23	0.520 ± 0.005	1.92 ± 0.02

^#^Standard deviation obtained from three independent experiments. *Not determined due to a low inhibition rate (<30% using 100 µM of the compound).

## Discussion

**Figure 6:**
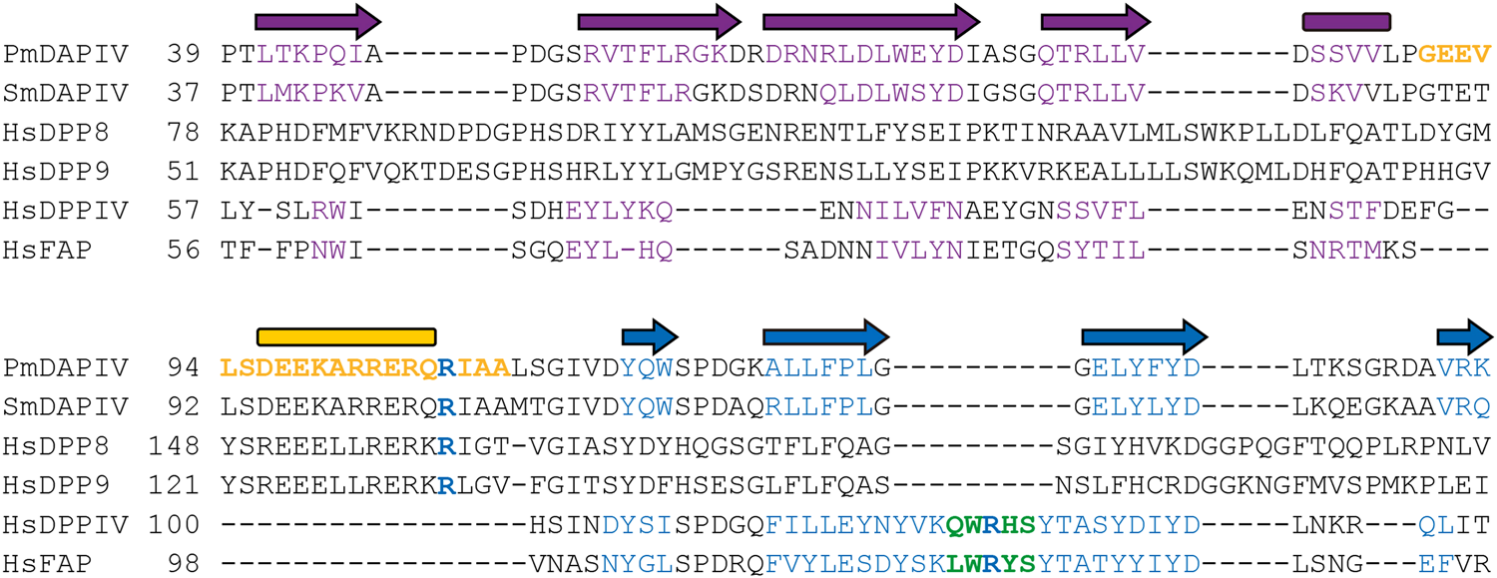
Amino acid sequences of the N-terminal region of DPP IV-family enzymes. Secondary structural elements of PmDAP IV are shown above the sequence alignment and those of each enzymes are coloured on the basis of their crystal structures, except for DPP8 and DPP9. A long insertion containing Arg106 located between blade-1 (purple) and blade-2 (blue) in PmDAP IV is shown in gold. A hairpin loop containing Arg125 located between strands 2 and 3 of blade-2 in human DPP IV is shown in green. The abbreviations used (UniProt accession numbers) are as follows: PmDAPIV (Q6F3I7), *Pseudoxanthomonas mexicana* DAP IV; SmDAPIV (P95782), *Stenotrophomonas maltophilia* DAP IV; HsDPP8 (Q6V1X1), *Homo sapiens* DPP8; HsDPP9 (Q86TI2), *Homo sapiens* DPP9; HsDPPIV (P27487), *Homo sapiens* DPP IV; and HsFAP (Q12884), *Homo sapiens* FAP.

In this study, we solved the crystal structures of a bacterial DPP IV, PmDAP IV, which specifically recognises a Pro/Ala residue at the P1 position of a substrate peptide. To better understand the substrate recognition mechanism of bacterial DAP IVs, we solved the structures of PmDAP IV in the presence of a peptide or an inhibitor. In the peptide complexes, the double-Glu motif, Glu208-Glu209, forms salt bridges to the N-terminus of the bound dipeptide. This interaction is essential for the exopeptidase activity of PmDAP IV. Interestingly, FAP has both dipeptidyl peptidase activity and endopeptidase activity^25,36^. Crystal structure of human FAP revealed one major difference in the vicinity of the double-Glu motif (Glu203-Glu204 for FAP) within the active site of the enzyme. Ala657 in FAP, instead of Asp663 as in human DPP IV, reduces the acidity in the N-terminus binding pocket, and this change could explain the lower affinity for N-terminal amines and endopeptidase activity of FAP^36^. For PmDAP IV, corresponding residue is Asp646 (Fig. 5), and this is consistent with our biochemical data that PmDAP IV has no endopeptidase activity^7^. The complex structures of PmDAP IV also revealed that the side chain of Arg106, which is located between blades 1 and 2 of the β-propeller domain, forms a hydrogen bond with the carbonyl group of the P2 residue of a substrate peptide and forms a salt bridge with the side chain of Glu208. In the ligand-free form of PmDAP IV and that of SmDAP IV^37^, a loop between blades 1 and 2 of the β-propeller domain, containing Arg106 in PmDAP IV, was disordered. The disordered loop region in the ligand-free form (residues 90–109 containing Arg106; Fig. 4) was well defined and formed a short helix in the dipeptide/inhibitor-bound complexes. The corresponding region in mammalian DPP IVs is replaced by a short turn and is not involved in substrate recognition. Site-directed mutagenesis studies of Arg106 in PmDAP IV (Table 3) indicated that the Arg side chain is essential for substrate recognition and for fixing the side chain of Glu208, one of the side chains in the double-Glu motif. Residues between blades 1 and 2 of the β-propeller domain, containing Arg106 in PmDAP IV, are highly conserved in SmDAP IV (Figure S6). In contrast, the region of Arg125 in human DPP IV is conserved in *P*. *gingivalis* DPP IV (Figure S6). Thus, DPP IVs can be categorised into two groups: human DPP IV-type DPP IVs and SmDAP IV-type DAP IVs. A phylogenetic analysis of several DPP IVs supports this notion (Figure S7). As shown in Figure S7, PmDAP IV and SmDAP IV belong to the same cluster, whereas PgDPP IV belongs to a bacterial cluster that is distinct from the SmDAP IV-type cluster but is related to a vertebrate DPP IV cluster. Interestingly, the phylogenetic analysis revealed that human dipeptidyl peptidase 8 (DPP8) and human dipeptidyl peptidase 9 (DPP9), which are ubiquitously expressed soluble DPP IV-like enzymes^25^ having approximately 25% amino acid sequence identities with human DPP IV, belong to the bacterial DAP IV cluster rather than the vertebrate DPP IV cluster. On the other hand, another DPP IV-like enzyme, human FAP^25^ having approximately 50% amino acid sequence identity with human DPP IV belongs to the vertebrate DPP IV cluster. To confirm the result of phylogenetic analysis on DPP8, DPP9 and FAP, we compared the amino acid sequence of PmDAP IV and those of the four DPP IV-family enzymes^25^ (Fig. 6). It is clear that the region of Arg106 in PmDAP IV is conserved in human DPP8 and human DPP9, whereas the region of Arg125 in human DPP IV is conserved in human FAP. Thus, it may be said that PmDAP IV is a bacterial DPP8/DPP9, though the Arg125-type DAP IV has not yet been identified in *P*. *mexicana*. Another interesting structural difference between human DPP IV and PmDAP IV is that human DPP IV has prominent exposed loops in the β-propeller domain than does PmDAP IV (Fig. 3). In particular, insertions Ala291-Met293 (between blade-4 and blade-5) and Val341-Ala342 (the fourth strand of blade-5) specifically found in human DPP IV (Figs 3 and S6) are involved in interactions with other proteins, such as adenosine deaminase^47^ and bat coronavirus HKU4 receptor-binding domain^48^.

**Figure 7:**
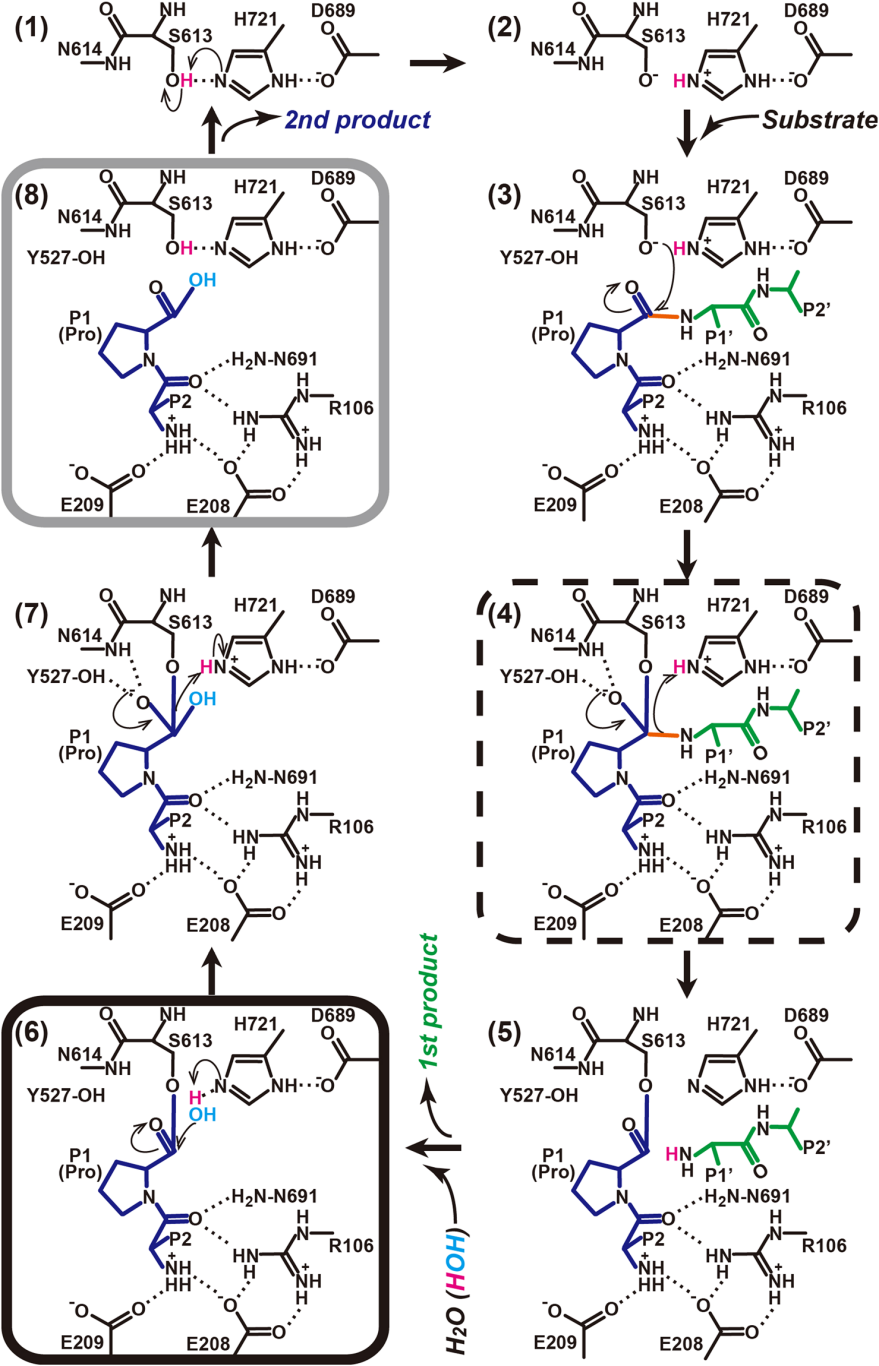
Possible reaction mechanism of the hydrolysis of a peptide substrate catalysed by PmDAP IV. Our crystal structures provide structural information for the sixth (the acyl-enzyme intermediate) and eighth (the second product) molecular stages of peptide digestion by PmDAP IV and are indicated by black and grey rounded rectangles, respectively. Human DPP IV complexed with diprotin A or with a decapeptide provides structural information for the fourth (the first tetrahedral intermediate) molecular stage and is indicated by a dashed-line rectangle.

The catalytic mechanism of serine peptidases involves two tetrahedral intermediates^49^. Figure 7 shows a possible catalytic mechanism of PmDAP IV, essentially identical to that of DPP IV^44^ except for the role of arginine side chain described later. The oxyanion-containing tetrahedral intermediate (Fig. 7, stages (4) and (7)) is stabilised by the backbone NH group of Asn614 and the side-chain OH group of Tyr527, which generate a positively charged pocket known as the oxyanion hole^44,50,51^. In the present crystal structure analyses, an acyl-enzyme intermediate (Fig. 7, stage (6)) is obtained by co-crystallisation of PmDAP IV with the tripeptide Lys-Pro-Tyr, and the N-terminal product, Lys-Pro, remains at the active site, where it is stabilised by salt bridges to the double-Glu motif Glu208-Glu209; finally, the C-terminal product, Tyr, dissociates from the active site. The hydrolysed dipeptide product NH_2_-Lys-Pro-COOH (Fig. 7, stage (8)) was also observed (Figure S1B). As can be seen from Figure 5A, the active site of acyl-intermediate complex of PmDAP IV has enough space near His721 to accommodate the nucleophilic water, although it could not be identified in the present crystal structure analyses. Interestingly, a similar acyl-enzyme intermediate is observed for PmDAP IV co-crystallised with the tripeptide, Ile-Pro-Ile, a well-known peptidyl DPP IV inhibitor, diprotin A^42,43^, resulting in a dipeptide Ile-Pro complex (Figure S1C).

In the complex of human DPP IV with the inhibitor diprotin A, a tetrahedral intermediate (Fig. 7, stage (4)), rather than an acyl-enzyme intermediate, was observed^34^. The bound diprotin A molecule is stabilised by hydrophobic interactions and a large network of salt bridges and hydrogen bonds. The N-terminus of the tripeptide interacts with the double-Glu motif (Glu205-Glu206), and the C-terminus interacts with Arg125 in human DPP IV. Notably, a non-covalent diprotin A complex has also been reported for human DPP IV^52^. In that case, Arg125 interacts with the carbonyl oxygen of the P1′-Ile residue and is responsible for fixing the conformation of the diprotin A molecule. Thus, the role of Arg125 is consistent in the covalent^34^ and the non-covalent^52^ diprotin A complexes in human DPP IV. A similar tetrahedral intermediate was reported for human DPP IV when complexed with the deca-peptide tNPY^39^. In the tNPY complex, a similar network of salt bridges and hydrogen bonds is observed. The carbonyl group of the third residue (P1′-residue) is stabilised by Arg125. Such an Arg-O = C interaction is present only for tripeptides or longer peptides and would prevent the release of leaving residues (P1′-P2′-) by stabilising the observed tetrahedral intermediate. In tripeptides or longer peptides, the negatively charged P1′ carboxylate (or carbonyl) group electrostatically stabilises the positively charged catalytic His740, thereby preventing transfer of its proton to the nitrogen atom on the released group (the N-terminus of the P1′ residue). Consequently, the C(P1)–N(P1′) bond cannot be cleaved, and the P1′ residue cannot leave from the active site of mammalian DPP IV^40^.

Interestingly, a structurally similar arginine residue, Arg106 in PmDAP IV, interacts with the carbonyl group of the P2 residue of the bound peptide. Therefore, Arg106 plays a role in stabilising the acyl-enzyme intermediate (Fig. 7, stage (6)), as observed in this study, rather than stabilizing the tetrahedral intermediate (Fig. 7, stage (4)), as observed for human DPP IV complexes^34,39,40^. To examine whether Arg106 in PmDAP IV is involved in recognition of the P1′-residue, we tested the inhibitory activity of diprotin A against the enzymatic activity of PmDAP IV (Table 5). The results showed that diprotin A is a good inhibitor of PmDAP IV. The comparable or stronger inhibitory activity of diprotin A against PmDAP IV, compared with that against human DPP IV (Table 5), indicates that the poorer interaction of the P1′ residue of diprotin A with PmDAP IV does not affect the inhibitory activity of diprotin A. Thus, the crystallographically observed covalently bonded interactions, the acyl-enzyme intermediate observed for PmDAP IV co-crystallised with diprotin A in this study (Figure S1C) and the tetrahedral intermediates observed for mammalian DPP IVs co-crystallised with diprotin A^34,39^ are essential structural factors related to the inhibitory activity of diprotin A.

**Table 5:** *K*_i_ and IC_50_ values of PmDAP IV and human DPP IV for diprotin A.

Enzyme	Residual activity (%)	*K*_i_ (µM)	IC_50_ (µM)
PmDAP IV wild-type	3.45 ± 0.10^#^	0.474 ± 0.020	2.91 ± 0.12
PmDAP IV R106A	7.63 ± 0.12	1.57 ± 0.26	7.41 ± 1.24
PmDAP IV R106K	8.17 ± 0.06	2.36 ± 0.11	9.19 ± 0.45
Human DPP IV	13.7 ± 0.8	4.01 ± 0.30	14.8 ± 1.1

^#^Standard deviation obtained from three independent experiments.

There are two possible routes to the active site of DPP IVs: the tunnel of the β-propeller domain and a side opening between the catalytic and β-propeller domains. For mammalian DPP IVs, it was proposed that the tunnel of the β-propeller and the side opening, respectively, provide substrate access to and product release from the active site of DPP IV^31^. However, crystal structure analyses of human DPP IV complexed with a decapeptide^39^ or a nonapeptide^53^ suggested that the side opening is the entrance to the active site because the bound peptides are oriented with the N-terminus facing the active site and with the C-terminus facing the side opening. MD simulations demonstrated that the side opening is more likely to be the pathway of product release than the β-propeller tunnel based on quantitative comparisons of force and work together with potentials of mean force between the two routes^54^. Thus, the side-in and side-out model is more plausible than the propeller-in and side-out model for mammalian DPP IVs.

In this crystal structure analysis of PmDAP IV, conformational differences were observed between the dipeptide- and an inhibitor-bound forms compared with the ligand-free form (Fig. 4). These observations are clearly different from the crystal structure analyses of mammalian DPP IVs, in which large-scale conformational differences have not yet been reported. It should be noted that an open form of a microbial prolyl oligopeptidase (POP), which shares a certain sequence homology to DPP IV, was reported^55^, such that large substrates could enter through the side opening. However, it is thought that POPs do not possess a DPP IV-equivalent side opening; thus, their internal cavity seems to be accessible only via the β-propeller tunnel^56^. The structures obtained here provide insights into the possible routes for substrate binding and product release for PmDAP IV. Regarding substrate access, the route from the tunnel of the β-propeller is unlikely. For the substrate to access the active site from the β-propeller tunnel, the peptide must adopt an energetically unfavourable, umbrella handle-shaped conformation, with the N-terminus facing the tip of the handle (towards the double-Glu motif) and with the C-terminus facing the body of the handle (in the tunnel) (Fig. 1B). In contrast, the access route from the side opening provides no impediment. For product release, egress from the side opening appears easy, but we cannot exclude the β-propeller tunnel route. In the dipeptide- or inhibitor-bound forms of PmDAP IV, the inter-domain cleft of PmDAP IV is relatively closed, and the bound peptide/inhibitor is buried in the active site and is not exposed to the solvent (Figs 1B and 5). The reaction product cannot exit from the active site without a closed-to-open conformational change. Thus, the product may exit from the active site (i) via the β-propeller tunnel without such a conformational change or (ii) via the side opening after such a conformational change. Finally, although the side-in and side-out model is highly possible, the side-in and propeller-out model cannot be excluded for bacterial DAP IVs. Notably, one of the four subunits (molecule B) in the asymmetric unit of the ligand-free crystal of PmDAP IV showed a relatively closed conformation, as observed for the dipeptide/inhibitor-bound complexes. The rms deviation between the molecule B of the ligand-free PmDAP IV and the molecule A of the Lys-Pro complex of PmDAP IV is 0.40 Å for all 724 Cα atoms. The disordered loop region in the molecules A, C and D of the ligand-free form (residues 90–109 containing Arg106) was well defined in the molecule B and the residues 96–105 formed a short helix as observed in the dipeptide/inhibitor complexes. The conformation around the short helix of the molecule B of the ligand-free form and that of the molecule A of the Lys-Pro complex is quite similar to each other (Figure S8), whereas relative flexibilities of the residues 90–109 estimated from temperature factors were significantly higher for the molecule B of the ligand-free form (average B-factor for whole molecule vs. that for residues 90–109: 36.3 Å^2^ vs 48.7 Å^2^ for the molecule B of the ligand-free form and 20.3 Å^2^ vs. 22.8 Å^2^ for the molecule A of the Lys-Pro complex). This observation indicates that an open-to-closed conformational change of the protomer of PmDAP IV does occur without ligand binding, and a mechanism of “conformational selection”^57,58^ would exist for the substrate-binding stage of PmDAP IV.

**Figure 8:**
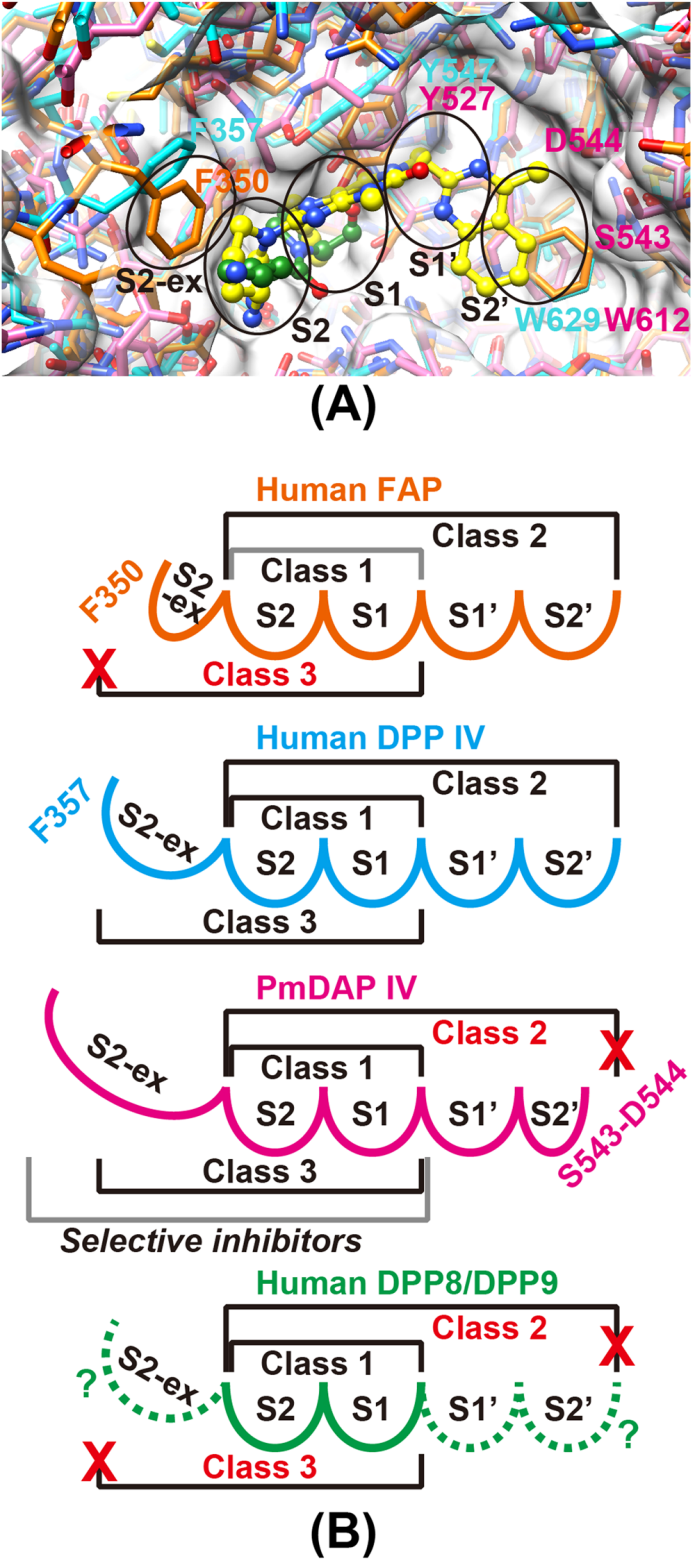
Active site cleft of PmDAP IV and human DPP IV-family enzymes. (**A**) Superposition of the active site of the Lys-Pro (green) complex of PmDAP IV (magenta) and those of the linagliptin (yellow) complex of human DPP IV (cyan) and ligand-free human FAP (orange). Subsites are indicated by ellipsoids. (**B**) A schematic figure showing the gliptin-binding sites of PmDAP IV and DPP IV-family enzymes. The diagrams of FAP, DPP IV, and PmDAP IV are drawn based on their crystal structure, whereas that of DPP8/DPP9 is a conceptual diagram.

To clarify the differences in active site architecture between PmDAP IV and mammalian DPP IV, we compared the inhibitory activities of the three classes of gliptins^46^ (Figure S5) against PmDAP IV and human DPP IV (Table 4). In general, the inhibitory activities of gliptins against human DPP IV (the order of nM) are overwhelmingly high compared to those against PmDAP IV (the order of µM). This is not unnatural because the structures of gliptins are optimised for binding with human DPP IV but not for bacterial DAP IVs. The difference might partly be due to less hydrophobic property at the bottom of the S1 subsite of PmDAP IV: Leu598 and Tyr631 in human DPP IV is replaced by Gln581 and Asn614, respectively, in PmDAP IV (Figure S7). The results showed that class 2 gliptins have significantly weaker inhibitory activities against PmDAP IV than class 1 and class 3 gliptins. It should be noted here that omarigliptin exhibited lower inhibitory activity against PmDAP IV as compared with the other class 3 gliptins. This can be explained by a structural feature of omarigliptin. As shown in Figure S5, omarigliptin has less rotatable bond than the other class 3 gliptins. Therefore, omarigliptin can’t fit well into the active site of PmDAP IV. The gliptin sensitivity of PmDAP IV can be explained by structurally comparing the subsites of PmDAP IV with that of human DPP IV in complex with linagliptin^59^ (Fig. 8A). Compared with human DPP IV, residues 542 to 545 of PmDAP IV protrude more into the S2′ subsite (Fig. 2), causing a narrowing of this site; thus, this region would hinder the binding of class 2 gliptins to PmDAP IV (Fig. 8B). In contrast to the S2′ subsite, the S2-extensive subsite of PmDAP IV is clearly larger than that of human DPP IV. The space occupied by residues 356 to 358 in human DPP IV is not occupied by protein residues in PmDAP IV (Fig. 8A), indicating that inhibitors occupying this space might be selective inhibitors of bacterial DAP IVs (Fig. 8B). A structural comparison of PgDPP IV and human DPP IV also revealed that the S2-extensive subsite of PgDPP IV is larger than that of human DPP IV^38^. Sitagliptin, a class 3 gliptin, is well-known as a highly selective DPP IV inhibitor^60^. Crystal structure analyses of human DPP IV in complexes with class 3 gliptins^61–64^ revealed that the most important interaction between gliptins and human DPP IV for their strict DPP IV selectivity (over DPP IV-family enzymes; FAP, DPP8 and DPP9) is a hydrophobic (pi-pi) interaction between the side chain of Phe357 and the inhibitor at the S2-extensive site. By utilising the S2 extensive subsite of human DPP IV, class 3 gliptins can increase not only their inhibitory activity against human DPP IV but also their selectivity over the other DPP IV-family enzymes. Specifically, PmDAP IV (and probably DPP8 and DPP9) cannot have sufficient hydrophobic interactions with the class 3 inhibitors at the S2-extensive site, whereas FAP does not have enough space at the S2-extensive site because Phe350 of FAP partially occupies the S2-extensive site (Fig. 8). Present structural comparisons can be applied for describing gliptin sensitivities of DPP8 and DPP9, though crystal structures of DPP8 and DPP9 have not yet been reported. DPP8 and DPP9 are known to be inhibited by class 1 gliptins (vildagliptin and saxagliptin) *in vitro*^60^, because the active site center (the S1 and S2 subsites) of DPP IV-family enzymes are highly conserved (Figure S7), except for Arg125 region (Figure S7). On the other hand, lengths and sequences around the S2′ and S2-extensive sites are less conserved as can be expected from lower sequence identity between DPP8/DPP9 and DPP IV (approximately 25%) compared with that between FAP and DPP IV (approximately 50%). Therefore, the lower sensitivities of DPP8/DPP9 against class 2 and 3 gliptins are likely to be due to structural differences around the S2′ and S2-extensive sites (Fig. 8B).

In this study, we present the crystal structures of a bacterial DPP IV, PmDAP IV, in its free form and in complexes with dipeptides as well as with a non-peptidyl inhibitor. The crystal structure analyses and site-directed mutagenesis studies revealed a novel substrate recognition mechanism, which is possibly common to SmDAP IV-type bacterial DPP IVs but is not observed in mammalian DPP IVs. Acyl-enzyme intermediates were observed for the dipeptide complexes of PmDAP IV, whereas tetrahedral intermediates were reported for the oligopeptide complexes of mammalian DPP IVs. This difference reflects the different structural environments of the active-site Arg residues of the mammalian and bacterial enzymes. We anticipate that the present structural analyses will support the design of specific inhibitors of the DPP IVs of pathogenic organisms.

## Methods

### Overexpression and purification of PmDAPIV

The periplasmic form of PmDAP IV was expressed and purified as described elsewhere^65^. A gene coding for PmDAP IV (residues 1–745) was cloned from a *P*. *mexicana* WO24 genomic DNA library by a cultivation plate assay based on DPP IV activity, the hydrolysis of Gly-Pro-β-naphthylamide^7^. The PmDAP IV M12I mutant produced only the 82-kDa periplasmic form (residues 23–745) due to translation from Met1 and removal of the signal sequence (residues 1–22)^11^. The periplasmic form of PmDAP IV comprised 723 amino acids, with a theoretical molecular weight of 79981.58 and a theoretical isoelectric point of 5.80. An *E*. *coli* JM109 (Takara Bio) transformant harbouring the full-length PmDAP IV M12I mutant sequence inserted into the pUC19 (Takara Bio) expression plasmid was used to produce the periplasmic form of PmDAP IV. Cells were grown in 2 x YT media at 310 K. Overproduction of PmDAP IV was performed by IPTG induction (final concentration, 0.1 mM) at an OD_600_ of approximately 0.6. Sixteen hours after induction, the cells were harvested by centrifugation at 8000 x g. The cells were disrupted using BugBuster Protein Extraction Reagent (Novagen), and the cell extract was obtained by centrifugation at 27,000 x g for 30 min. PmDAP IV in the cell extract was purified using 35 to 70% ammonium sulphate precipitation and hydrophobic column chromatography using a 20-ml HiPrep 16/10 Butyl column (GE Healthcare), which was desalted using a 50-ml HiPrep 26/10 desalting column (GE Healthcare) and finally subjected to anion-exchange column chromatography using a 1-ml Mono Q 5/50 GL column (GE Healthcare). The fractions containing PmDAP IV were pooled, and the buffer was exchanged to 80 mM Tris-HCl pH 8.5 and concentrated to 10 mg/ml using a Vivaspin 20 concentrator (GE Healthcare). The protein concentration was determined using the Bradford assay and a bovine serum albumin as a standard (Bio-Rad Laboratories). The column chromatography and other purification steps were performed at 298 K and 277 K, respectively.

### Crystallisation

To obtain ligand-free PmDAP IV crystals, the samples were crystallised using the hanging-drop method; 1 μl of protein solution (10 mg/ml PmDAP IV in 80 mM Tris-HCl, pH 8.5) was mixed with the same volume of reservoir solution (20%(v/v) glycerol, 9.6%(m/v) PEG20000 and 80 mM MES pH 6.5) and incubated at 293 K. The drops were suspended over 200 μl of reservoir solution in 48-well plates. Crystals of the PmDAP IV/dipeptide (tripeptides were used as a substrate, and the N-terminal dipeptide was observed as the bound product) or inhibitor complexes were prepared as follows: Lys-Pro-Tyr, Ile-Pro-Ile (diprotin A) and 1-({[1-(hydroxymethyl)cyclopentyl]amino}-acetyl)pyrrolidine-2,5-cis-dicarbonitrile (Inhibitor-1c) were purchased from Bachem AG (Bubendorf, Switzerland), Peptide Institute, Inc. (Osaka, Japan), and Wako Pure Chemical Industries, Ltd. (Osaka, Japan), respectively. The tripeptides Lys-Pro-Tyr and Ile-Pro-Ile and the inhibitor were dissolved in 80 mM Tris-HCl pH 8.5 to concentrations of 40.0 mM, 30.0 mM and 40.5 mM, respectively. The 10-mg/ml PmDAP IV solution was mixed with aliquots of the respective ligand solutions at volume ratios of 9:1, 4.5:1 and 26:1 for the tripeptides Lys-Pro-Tyr and Ile-Pro-Ile and the inhibitor, respectively, with final ligand concentrations of 4 mM, 5.5 mM and 1.5 mM, respectively. A droplet was prepared by mixing an equal volume (1 μl + 1 μl) of the working solution described above and the reservoir solution containing 20%(v/v) glycerol and 12%(m/v) PEG20000 in 80 mM MES buffer at pH 6.5 for the tripeptide complexes and 20%(v/v) glycerol and 12%(m/v) PEG20000 in 100 mM MES buffer at pH 6.5 for the inhibitor complex. The droplet was suspended over 500 μl of reservoir solution in each well of a 24-well plate.

### X-ray data collection

Because the crystallisation conditions of the ligand-free and dipeptide/inhibitor-bound PmDAP IVs (described above) included 20%(v/v) glycerol in the reservoir solution, X-ray data could be collected under cryogenic conditions without the need for any additional cryoprotectant. Crystals obtained using the hanging-drop method were directly mounted in nylon loops and flash-cooled in a cold nitrogen gas stream at 100 K immediately before data collection. Data were collected by the rotation method at 100 K using ADSC Quantum CCD detectors or a PILATUS-2M detector with synchrotron radiation sources at the Photon Factory. Laue group and unit-cell parameters were determined using the XDS software package^66^. The resulting cell parameters and data-collection statistics are summarised in Table 1.

### Structure determination and refinement

The initial phase determination was performed using the molecular replacement method and one protomer of SmDAP IV^37^ (PDB code: 2ECF) as the search model. Cross-rotation and translation functions were calculated using the programme MOLREP^67^ from the CCP4 suite^68^. Automatic model building and refinement were carried out using the programs ARP/wARP^69^ and REFMAC5^70^, and further iterative manual model building and refinement were performed using the programs Coot^71^ and REFMAC5. The stereochemistry of the model was verified using the program RAMPAGE^72^. The refined structure of the ligand-free PmDAP IV was then used for the structural determination of the dipeptide or inhibitor complexes by the difference Fourier method. The occupancies of ligand atoms of ligand complexes were estimated by varying the occupancies of ligand atoms set at 0.60 to 0.95, in increments of 0.05. The results showed that the temperature factors of P1-Pro became closer to those of the surrounding atoms, without producing a positive residual peak in the |*F*o|−|*F*c| map, when the occupancies of the ligand atoms were set at 0.90. Occupancies below 0.90 resulted in positive residual peaks in the |*F*o|−|*F*c| map for the bound ligand atoms. After the final round of refinement, the ligand molecule and the Oγ atom of Ser613 were removed from the model. Then, the amplitude |*F*c| and phase angles calculated from the partial structure were used to calculate a weighted *m*|*F*o|–*D*|*F*c| omit map^70^, where *m* is the figure of merit (approximately equal to the cosine of the phase error) and *D* is the estimate of the coordinate error in the partial structure (Figure S1). The refinement statistics are summarised in Table 2.

### Enzymatic activity assays of wild-type and mutant PmDAP IVs and human DPP IV

Arg106 of PmDAP IV, which is involved in the recognition of the carbonyl group of the P2 residue of the substrate peptide, was mutated to Ala or Lys. The mutants were expressed and purified using the method described above for wild-type PmDAP IV. Recombinant human DPP IV was purchased from ATGen Co., Ltd. (Los Angeles, USA). Kinetic parameters were determined by fitting the experimental data to the Michaelis–Menten equation using Excel Solver (Microsoft) by nonlinear least squares fitting for different concentrations (0.78, 1.56, 3.13, 6.25, 12.5, 25, 50 and 100 µM) of glycyl-L-proline 4-methylcoumaryl-7-amide (Gly-Pro-MCA, Peptide Institute) as a substrate. The enzyme reaction was performed in a reaction buffer containing 50 mM sodium phosphate buffer pH 8.0, 5 mM EDTA and 0.005% Tween 20 at 298 K for 20 min, and standard deviations were calculated from three independent experiments. The fluorescence intensity of the released MCA was measured with excitation at 355 nm and emission at 460 nm using an Infinite 200 PRO microplate reader (TECAN). The relative activities of the wild-type and mutant PmDAP IVs and human DPP IV are summarised in Table 3.

### Inhibitor sensitivities of PmDAP IV and human DPP IV

Gliptins and diprotin A were purchased from NAMIKI SHOJI Co., Ltd. (Tokyo, Japan) and Peptide Institute, Inc. (Osaka, Japan), respectively. Half-maximal inhibitory concentration (IC_50_) values were determined by fitting to a sigmoid curve (4-parameter logistic curve) using ImageJ^73^. Competitive inhibition rates were measured using different concentrations of each inhibitor in a reaction buffer containing 50 mM sodium phosphate buffer pH 8.0, 5 mM EDTA and 0.005% Tween 20 at 298 K for 20 min and using 100 µM Gly-Pro-MCA as the substrate. Inhibitor concentrations for PmDAP IV were 0.78, 1.56, 3.13, 6.25, 12.5, 25, 50 and 100 µM, and those for human DPP IV were 0.78, 1.56, 3.13, 6.25, 12.5, 25, 50 and 100 nM. Standard deviations were calculated from three independent experiments. Fluorescence intensity was measured with excitation at 355 nm and emission at 460 nm using an Infinite 200 PRO microplate reader. Inhibition constants (*K*_i_) were calculated using the Cheng-Prusoff equation^74^. Gliptin sensitivities and diprotin A sensitivities of the wild-type and mutant PmDAP IVs and human DPP IV are summarised in Tables 4 and 5, respectively.

### Graphical programs

Figures 1 to 5, 8, S1, S3 and S8 were produced using the programmes UCSF Chimera^75^ and Adobe Illustrator (Adobe Systems Inc., San Jose, California, USA). Figures 6, 7 and S2, S4 to S7 were produced using Adobe Illustrator.

### Accession codes

Atomic coordinates for the reported structures have been deposited in the Protein Data Bank under accession codes 5YP1 (free form), 5YP2 (Inhibitor-1c complex), 5YP3 (Ile-Pro complex) and 5YP4 (Lys-Pro complex).

## Electronic supplementary material

Supplementary Information
