# Supplementary material for: Crystal structures of a bacterial dipeptidyl peptidase IV reveal a novel substrate recognition mechanism distinct from that of mammalian orthologues
Source: Sci Rep. 2018 Feb 9;8:2714. doi: 10.1038/s41598-018-21056-y (PMC5807507; doi:10.1038/s41598-018-21056-y)
Supplement: Supplementary file 1 — Supplementary Information [file 41598_2018_21056_MOESM1_ESM.pdf]

SUBJECT AREAS:

ENZYME MECHANISMS, PROTEASES, STRUCTURAL BIOLOGY

*Correspondence and requests for materials should be addressed to
N.T. (ntanaka@pharm.showa-u.ac.jp) or W.O. (owataru@vos.nagaokaut.ac.jp)

Crystal structures of a bacterial dipeptidyl peptidase IV reveal a novel substrate recognition mechanism distinct from that of mammalian orthologues

Saori Roppongi[1], Yoshiyuki Suzuki[2], Chika Tateoka[1], Mayu Fujimoto[1], Saori Morisawa[1], Ippei Iizuka[1], Akihiro Nakamura[2], Nobuyuki Honma[2], Yosuke Shida[2], Wataru Ogasawara[2,*], Nobutada Tanaka[3,*], Yasumitsu Sakamoto[1], and Takamasa Nonaka[1]

[1]School of Pharmacy, Iwate Medical University, 2-1-1 Nishitokuta, Yahaba, Iwate 028-3694, Japan; [2]Department of Bioengineering, Nagaoka University of Technology, 1603-1 Kamitomioka, Nagaoka, Niigata 940-2188, Japan; [3]School of Pharmacy, Showa University, 1-5-8 Hatanodai, Shinagawa-ku, Tokyo 142-8555, Japan

Supplementary information includes:

Supplementary Figures S1-S8

Figure S1

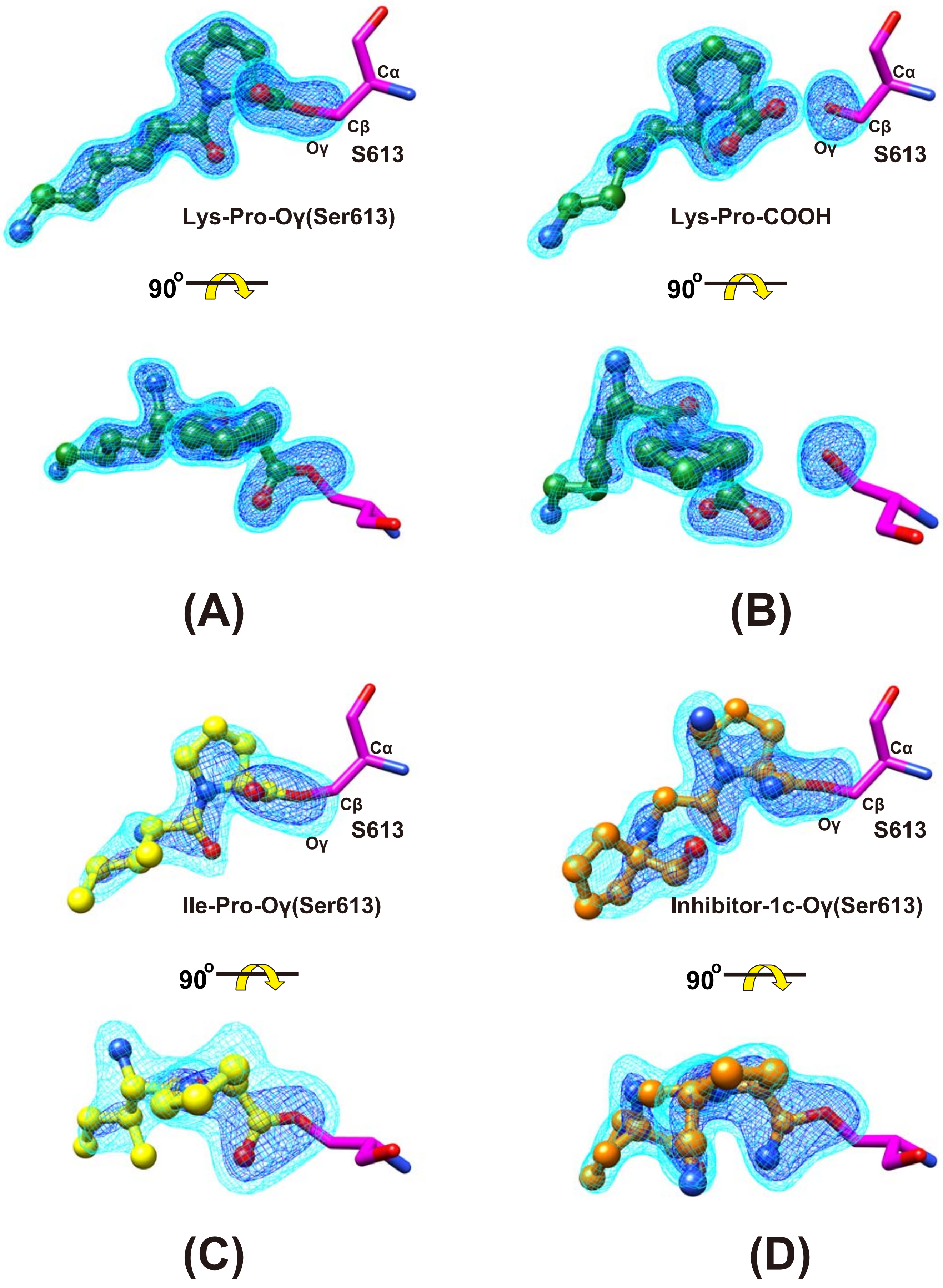

Cα
Cβ
Oγ
S613
Lys-Pro-Oγ(Ser613)
Cα
Cβ
Oγ
S613
Lys-Pro-COOH
90°
90°
(A)
(B)
Cα
Cβ
S613
Oγ
Ile-Pro-Oγ(Ser613)
Cα
Cβ
Oγ
S613
Inhibitor-1c-Oγ(Ser613)
90°
90°
(C)
(D)

Figure S2

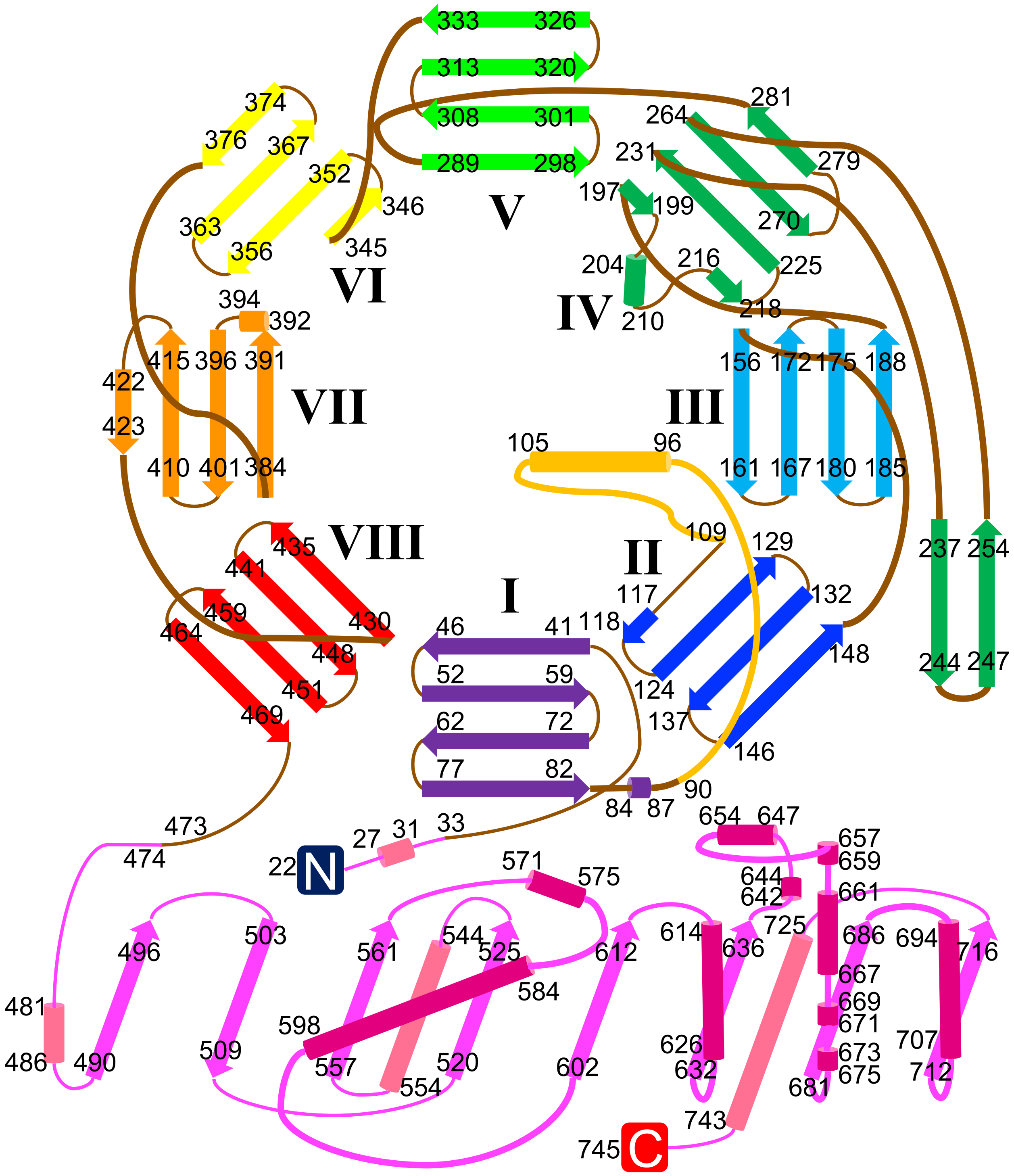

333
326
313
320
308
301
289
298
281
264
279
231
270
197
199
225
204
216
218
210
374
376
367
352
346
363
356
345
V
VI
IV
394
392
415
396
391
422
423
410
401
384
VII
III
156
172
175
188
161
167
180
185
105
96
109
129
117
132
118
148
124
137
146
237
254
244
247
II
I
VIII
435
441
430
459
464
448
451
469
46
41
52
59
62
72
77
82
84
87
90
473
474
27
31
33
22
N
654
647
657
659
644
642
661
571
575
481
486
490
496
503
509
561
544
525
598
557
554
520
584
612
602
614
636
626
632
725
686
694
716
667
669
671
673
675
681
707
712
743
745
C

Figure S3

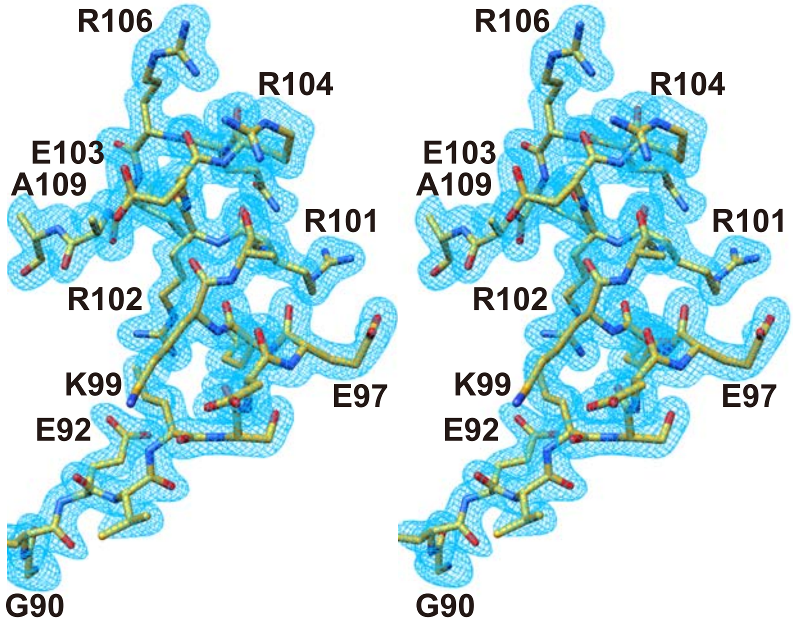
R106
R104
E103
A109
R101
R102
K99
E97
E92
G90
R106
R104
E103
A109
R101
R102
K99
E97
E92
G90

Figure S4

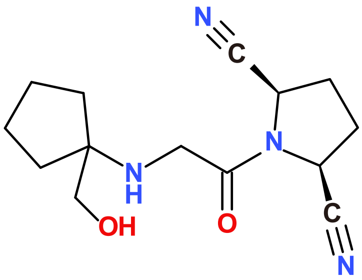

Inhibitor-1c

Figure S5

Class 1

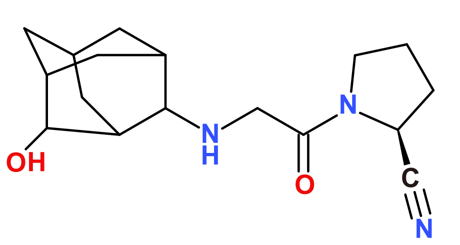

Vildagliptin

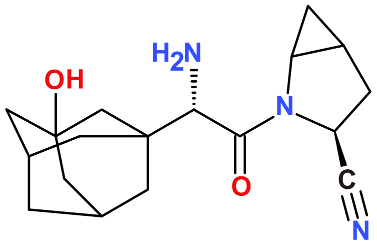

Saxagliptin

Class 2

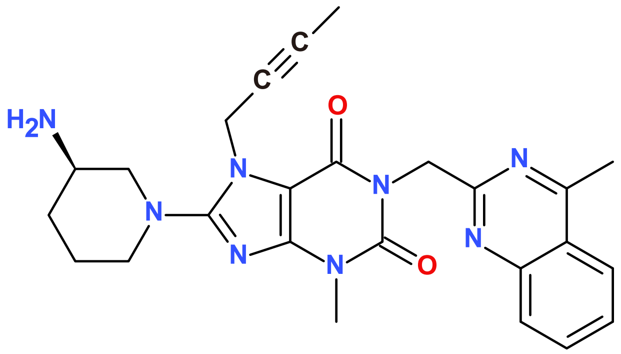

Linagliptin

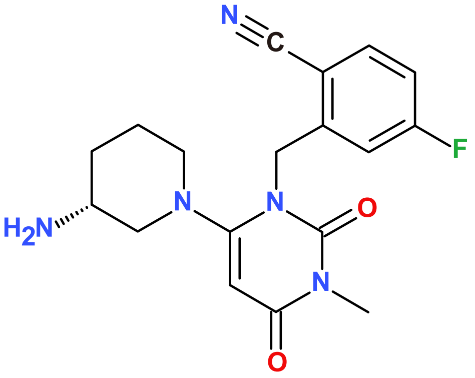

Trelagliptin

Class 3

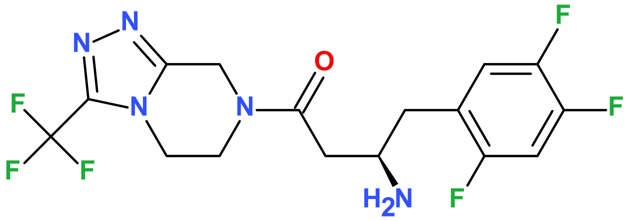

Sitagliptin

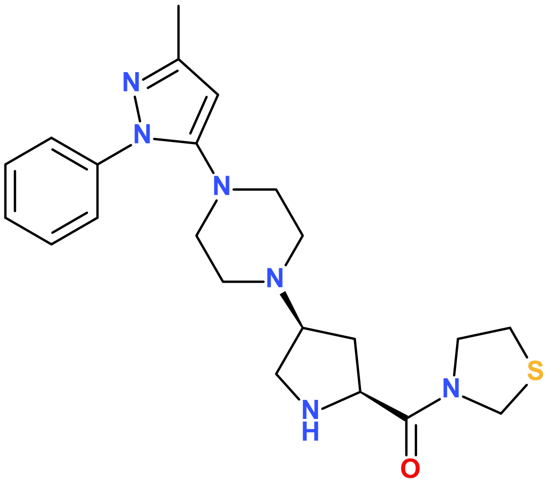

Teneligliptin

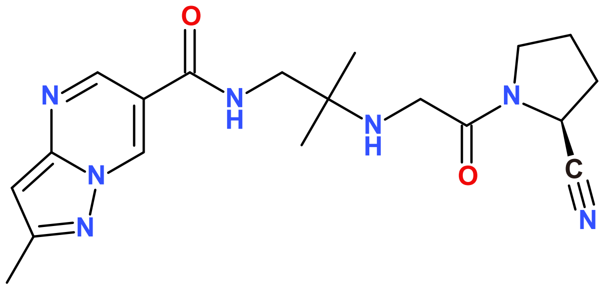

Anagliptin

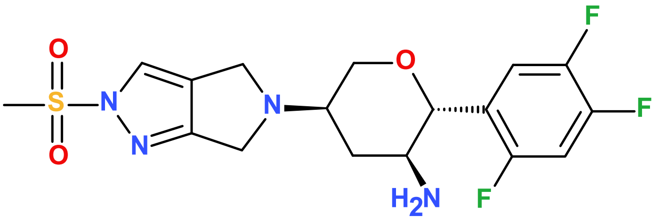

Omarigliptin

Figure S6

```
PmDAPIV 19  PAHAEKLTLE AITGSAPLSG PTL-TKPQIA PD-GSRVTFL RGKDRDRNRL DLWEYDIASG -QTRLLVDSS VVLPGEEVLS DEEKARRERQ RIAALSGIVD
SmDAPIV 17  VAHAEKLTLE AITGPLPLSG PTL-MKPKVA PD-GSRVTFL RGKDSDRNQL DLWSYDIGSG -QTRLLVDSK VVLPGTETLS DEEKARRERQ RIAAMTGIVD
HsDPPIV 38  DSRKTYTLTD YLKNT-YRLK LY--SLRWI- SD-HEYLYKQ --------EN NILVFNAEYG -NSSVFLENS TFDEFG---- ---------- -----HSIND
PgDPPIV 21  TGDKPVDLKE ITSGM-FYAR SAGRGIRSM- PDGEHYTEMN RE------RT AIVRYNYASG KAVDTLFSIE RARECPF--- ---------- -----KQIQN

PmDAPIV 116 YQWSPDGKAL LFPLG----- -----GELYF YDLTKSGRDA VRK-LTNGGG FATDPKISPK GGFVSFIRDR NLWAIDLASG KEVQLTRDGS D-TIGNGVAE
SmDAPIV 114 YQWSPDAQRL LFPLG----- -----GELYL YDLKQEGKAA VRQ-LTHGEG FATDAKLSPK GGFVSFIRGR NLWVIDLASG RQMQLTADGS T-TIGNGIAE
HsDPPIV 105 YSISPDGQFI LLEYNYVKQW RHSYTASYDI YDLNKR---Q LITEERIPN- NTQWVTWSPV GHKLAYVWNN DIYVKIEPNL PSYRITWTGK EDIIYNGITD
PgDPPIV 95  YEVSSTGHHI LLFTDMESIY RHSYRAAVYD YDVRRN---L VKPLSEHVG- KVMIPTFSPD GRMVAFVRDN NIFIKKFDFD TEVQVTTDGQ INSVLNGATD

PmDAPIV 204 FVADEEMDR- HTGYWWAPDD AAIAFARIDE TPVPVQKRYE VYP---DRTE VVEQRYPAAG DHNVRVQLGV IAPKT----- GARPRWIDLG ---KDPDIYL
SmDAPIV 202 FVADEEMDR- HTGYWWAPDD SAIAYARIDE SPVPVQKRYE VYA---DRTD VIEQRYPAAG DANVQVKLGV ISPAE----- QAQTQWIDLG ---KEQDIYL
HsDPPIV 201 WVYEEEVFSA YSALWWSPNG TFLAYAQFND TEVPLIEYSF YSDESLQYPK TVRVPYPKAG AVNPTVKFFV VNTDSLSSVT NATSIQITAP ASMLIGDHYL
PgDPPIV 191 WVYEEEFGV- TNLMSWSADN AFLAFVRSDE SAVPEYRMPM YED--KLYPE DYTYKYPKAG EKNSTVSLHL YNVAD----- -RNTKSVSLP ---IDADGYI

PmDAPIV 292 ARVDWRD-PQ RLTFQRQSRD QKKIELIETT LT----NGTQ R--TLVTETS T-TWV----P LHNDLRFLK- DGRFLWSSER S-GFEHLYVA SEDGSTLTAL
SmDAPIV 290 ARVNWRD-PQ HLSFQRQSRD QKKLDLVEVT LA----SNQQ R--VLAHETS P-TWV----P LHNSLRFLD- DGSILWSSER T-GFQHLYRI DS-KGKAAAL
HsDPPIV 301 CDVTWAT-QE RISLQWLRRI QNYSVMDICD YDESSGRWNC LVARQHIEMS TTGWVGRFRP -SEP-HFTLD GNSFYKIISN EEGYRHICYF QIDKKDCTFI
PgDPPIV 279 PRIAFTDNAD ELAVMTLNRL QNDFKMYYVH PK----SLVP K--LILQDMN K-RYV-DSDW IQAL-KFTA- GGGFAYVSEK D-GFAHIYLY DNKGVMHRRI

PmDAPIV 378 TQGEWVVDSL LAIDEAAGLA YVSGTRD--G ATEAHVYAVP LS-GGEPRRL TQA-----PG MHAATFARNA SVFVDSWSSD TTLPQIELFK AD-GTKLATL
SmDAPIV 375 THGNWSVDEL LAVDEKAGLA YFRAGIE--S ARESQIYAVP LQ-GGQPQRL SKA-----PG MHSASFARNA SVYVDSWSNN STPPQIELFR AN-GEKIATL
HsDPPIV 398 TKGTWEVIGI EALTS--DYL YYISNEYKGM PGGRNLYKIQ LSDYTKVTCL SCELNPERCQ YYSVSFSKEA KYYQLRCSGP -GLPLYTLHS SVNDKGLRVL
PgDPPIV 368 TSGNWDVTKL YGVDA-SGTV FYQSAEE--S PIRRAVYAID AK--GRKTKL SLN-----VG TNDALFSGNY AYYINTYSSA ATPTVVSVFR SKGAKELRTL

PmDAPIV 469 LVND-VSDAT HPYAKYRAAH QPTAYGTLTA ADGTTPLHYS LIKPAGFDPK KQYPVVVFVY GGPAAQTVTR AWPGRSDSFF NQYLAQ-QGY VVFTLDNRGT
SmDAPIV 466 VEND-LADPK HPYARYREAQ RPVEFGTLTA ADGKTPLNYS VIKPAGFDPA KRYPVAVYVY GGPASQTVTD SWPGRGDHLF NQYLAQ-QGY VVFSLDNRGT
HsDPPIV 495 EDNSALDKM- --LQNV--QM PSKKLDFIIL --NETKFWYQ MILPPHFDKS KKYPLLLDVY AGPCSQKADT VFRL----NW ATYLASTENI IVASFDGRGS
PgDPPIV 458 EDNVALRER- --LKAY--RY NPKEFTIIKT QS-ALELNAW IVKPIDFDPS RHYPVLMVQY SGPNSQQVLD RYSF----DW EHYLAS-KGY VVACVDGRGT

PmDAPIV 567 PRRGAAFGGA LYGKQGTVEV DDQLRGIEWL KSQAFVDPAR IGVYGWSNGG YMTLMLLAKH DEAYACGVAG APVTDWALYD THYTERYMDL PK--ANEAGY
SmDAPIV 564 PRRGRDFGGA LYGKQGTVEV ADQLRGVAWL KQQPWVDPAR IGVQGWSNGG YMTLMLLAKA SDSYACGVAG APVTDWGLYD SHYTERYMDL PA--RNDAGY
HsDPPIV 584 GYQGDKIMHA INRRLGTFEV EDQIEAARQF SKMGFVDNKR IAIWGWSYGG YVTSMVLGSG SGVFKCGIAV APVSRWEYYD SVYTERYMGL PTPEDNLDHY
PgDPPIV 547 GARGEEWRKC TYMQLGVFES DDQIAAATAI GQLPYVDAAR IGIWGWSYGG YTTLMSLCRG NGTFKAGIAV APVADWRFYD SVYTERFMRT PK--ENASGY

PmDAPIV 665 REASVFTHVD GIGAGKLLLI HGMADDNVLF TNSTKLMSEL QKRGTPFELM TYPGAKHGLR -GSDLLHRYR LTEDFFARCL KP
SmDAPIV 662 REARVLTHIE GL-RSPLLLI HGMADDNVLF TNSTSLMSAL QKRGQPFELM TYPGAKHGLS -GADALHRYR VAEAFLGRCL KP
HsDPPIV 684 RNSTVMSRAE NFKQVEYLLI HGTADDNVHF QQSAQISKAL VDVGVDFQAM WYTDEDHGIA SSTAHQHIYT HMSHFIKQCF SLP
PgDPPIV 645 KMSSALDVAS QL-QGNLLIV SGSADDNVHL QNTMLFTEAL VQANIPFDMA IYMDKNHSIY GGNTRYHLYT RKAKFLFDNL
```

Figure S7

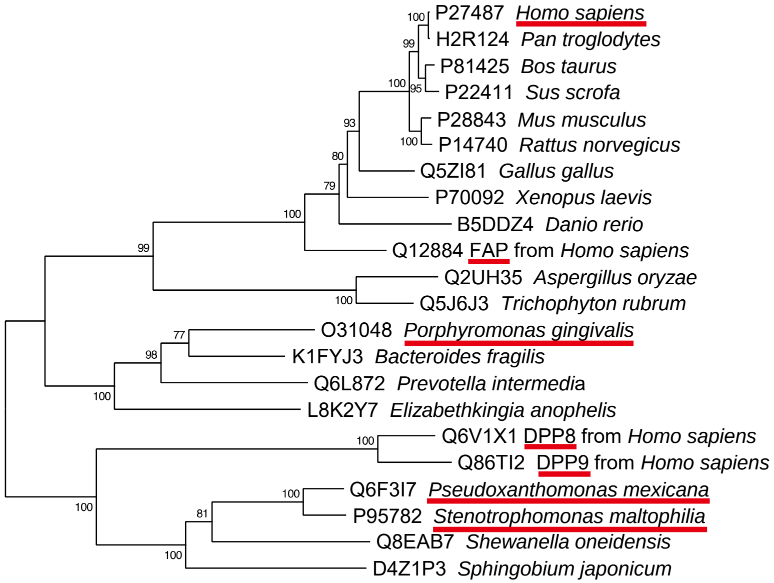

(A)

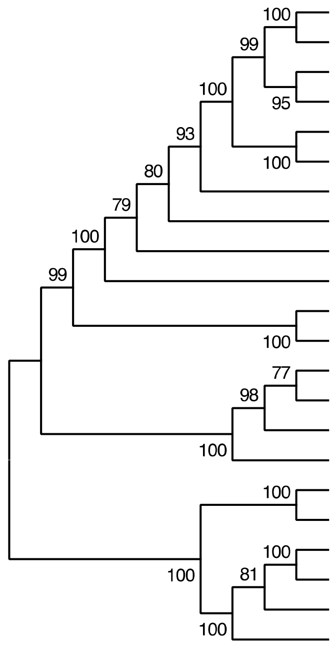

Phylogenetic tree	Recognition sites of family S9	S2 pocket					S1 pocket											S1' pocket	S2' pocket					
	Site number of DAPIV from *P. mexicana*	106	*125**	208	209	*357**	527	531	581	614	639	642	645	646	649	691	692	532	542	543	544	612	722	731
P27487	*Homo sapiens*	-	R	E	E	F	Y	C	L	Y	V	W	Y	D	Y	N	V	S	-	-	-	W	G	I
H2R124	*Pan troglodytes*	-	R	E	E	F	Y	C	L	Y	V	W	Y	D	Y	N	V	S	-	-	-	W	G	I
P81425	*Bos taurus*	-	R	E	E	F	Y	C	L	Y	V	W	Y	D	Y	N	V	S	-	-	-	W	G	I
P22411	*Sus scrofa*	-	R	E	E	F	Y	C	L	Y	V	W	Y	D	Y	N	V	S	-	-	-	W	G	I
P28843	*Mus musculus*	-	R	E	E	F	Y	C	L	Y	V	W	Y	D	Y	N	V	S	-	-	-	W	G	I
P14740	*Rattus norvegicus*	-	R	E	E	F	Y	C	L	Y	V	W	Y	D	Y	N	V	S	-	-	-	W	G	I
Q5ZI81	*Gallus gallus*	-	R	E	E	I	Y	C	L	Y	V	W	Y	D	Y	N	V	S	-	-	-	W	G	I
P70092	*Xenopus laevis*	-	R	E	E	F	Y	G	L	Y	V	W	Y	D	Y	N	V	S	-	-	-	W	G	I
B5DDZ4	*Danio rerio*	-	R	E	E	F	Y	C	L	Y	V	W	Y	D	Y	N	V	S	-	-	-	W	S	V
Q12884	FAP from *Homo sapiens*	-	R	E	E	F	Y	C	L	Y	V	W	Y	A	Y	N	V	S	-	-	-	W	G	L
Q2UH35	*Aspergillus oryzae*	-	R	E	E	M	Y	G	L	F	V	W	Y	D	Y	N	V	A	-	-	-	W	G	L
Q5J6J3	*Trichophyton rubrum*	-	R	E	E	-	Y	G	L	Y	V	F	Y	D	Y	N	V	A	-	-	-	X	G	Q
O31048	*Porphyromonas gingivalis*	-	R	E	E	D	Y	N	L	Y	V	W	Y	D	Y	N	V	S	-	-	-	W	S	L
K1FYJ3	*Bacteroides fragilis*	-	R	E	E	N	Y	G	L	F	P	W	Y	D	Y	N	V	S	-	-	-	W	G	L
Q6L872	*Prevotella intermedia*	-	R	E	E	D	Y	G	L	F	P	W	Y	D	Y	N	V	S	-	-	-	W	S	L
L8K2Y7	*Elizabethkingia anophelis*	-	R	E	E	D	Y	G	L	F	V	W	Y	D	Y	N	V	S	-	-	-	W	G	L
Q6V1X1	DPP8 from *Homo sapiens*	R	-	E	E	F	Y	Q	M	Y	V	W	Y	D	Y	N	V	V	-	-	-	W	S	Y
Q86TI2	DPP9 from *Homo sapiens*	R	-	E	E	F	Y	Q	M	Y	V	W	Y	D	Y	N	V	V	-	-	-	W	S	Y
Q6F3I7	*Pseudoxanthomonas mexicana*	R	-	E	E	-	Y	A	Q	N	V	W	Y	D	Y	N	V	A	R	S	D	W	G	R
P95782	*Stenotrophomonas maltophilia*	R	-	E	E	-	Y	A	Q	N	V	W	Y	D	Y	N	V	S	R	G	D	W	G	R
Q8EAB7	*Shewanella oneidensis*	R	-	E	E	-	Y	H	L	Y	V	W	Y	D	Y	N	V	A	E	Q	D	H	S	L
D4Z1P3	*Sphingobium japonicum*	R	-	E	E	-	Y	G	M	Y	V	W	Y	D	Y	N	V	A	-	-	-	W	R	L

(B)

Figure S8

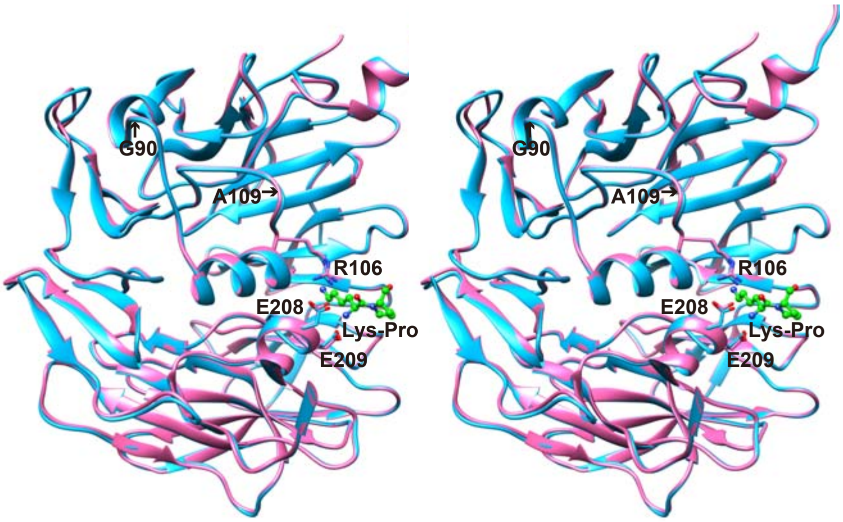
G90
A109
R106
E208
Lys-Pro
E209
G90
A109
R106
E208
Lys-Pro
E209

Figure S1 | Weighted *m*|*F*o| - *D*|*F*c| omit maps of the bound dipeptide/inhibitor in the active site of PmDAP IV. The contour levels are 3.0 σ (cyan) and 5.0 σ (blue). The carbon atoms of Ser613 in PmDAP IV are coloured magenta. (A) Lys-Pro (green) is observed as an acyl-enzyme intermediate in subunit A at 1.90-Å resolution. (B) Lys-Pro (green) is observed as a product containing a complete C-terminus in subunit C at 1.90-Å resolution. (C) Ile-Pro (yellow) is observed as an acyl-enzyme intermediate in subunit A at 2.44-Å resolution. (D) Inhibitor-1c (orange) is observed as a covalently bound form at 2.13-Å resolution.

Figure S2 | Topology diagram of PmDAP IV. α-Helices and β-strands are shown as cylinders and arrows, respectively. The catalytic domain is coloured in magenta. The β -propeller domain is coloured from purple (blade 1) to red (blade 8). A long insertion containing Arg106 located between blade-1 and blade-2, which was disordered in the ligand-free form, is shown in gold.

Figure S3 | Wall-eyed stereo diagram showing a weighted *m*|*F*o| - *D*|*F*c| omit map of residues 90-109 in the β-propeller domain of PmDAP IV (Lys-Pro complex) at 1.90 Å. The contour level is 3.0 σ.

Figure S4 | The structural formula of Inhibitor-1c used in this study.

Figure S5 | The structural formula of gliptins used in this study.

Figure S6 | Amino acid sequences of DPP IVs. The catalytic (red), P2-main chain recognition (blue), P1-side chain recognition (green) and S2-extensive site (magenta) residues are highlighted. The abbreviations used are as follows: PmDAPIV, *Pseudoxanthomonas mexicana* DAP IV; SmDAPIV, *Stenotrophomonas maltophilia* DAP IV; PgDPPIV, *Porphyromonas gingivalis* DPP IV; and HsDPPIV, *Homo sapiens* DPP IV. Secondary structural elements are shown above the sequence alignment. The catalytic domain is coloured in magenta. The β-propeller domain is coloured from purple (blade 1) to red (blade 8). An insertion helix located between blade-1 and blade-2, which was disordered in the ligand-free form, is shown in gold. Insertions of human DPP IV involved in interactions with other proteins are shown in orange.

Figure S7 | Molecular phylogenetic analysis and substrate recognition residues of DPP IVs. The phylogenetic tree was constructed by MUSCLE multiple sequence alignment and using the maximum likelihood method based on the Le_Gascuel_2008 model. The percentage of replicate trees in which the associated taxa clustered together in the bootstrap test (1000 replicates) are shown next to the branches. Initial trees for the heuristic search were obtained automatically by applying Neighbour-Join and BioNJ algorithms to a matrix of pairwise distances estimated using a JTT model and then selecting the topology with the superior log likelihood value. A discrete Gamma distribution was used to model evolutionary rate differences among sites (5 categories (+G, parameter = 2.1947)). The rate variation model allowed for some sites to be evolutionarily invariable ([+I], 3.43% sites). All positions containing gaps and missing data were eliminated. In total, 639 positions were present in the final data set. Evolutionary analyses were conducted in MEGA7. Enzymes referred to in the text are underlined. (A) A phylogenetic tree showing branch lengths measured as the number of substitutions per site. (B) Topology phylogenetic tree and substrate recognition residues of the subsites S2

to S2'. Asterisked numbers correspond to the residue numbers of human DPP IV because the residues are missing in PmDAP IV. The position 612 of DPP IV from *Trichophyton rubrum* is registered as "X" in the UniProt sequence ID Q5J6J3.

Figure S8 | Wall-eyed stereo view showing a structural comparison of the β-propeller domain of the molecule B of the ligand-free form (blue) of PmDAP IV and that of the molecule A of the Lys-Pro complex (pink) of PmDAP IV. The side chains of Arg106, Glu208, and Glu209 are shown as stick models. The bound Lys-Pro molecule (green) is shown as a ball-and-stick model.
